# Proposed guidelines for the diagnosis and management of methylmalonic and propionic acidemia

**DOI:** 10.1186/s13023-014-0130-8

**Published:** 2014-09-02

**Authors:** Matthias R Baumgartner, Friederike Hörster, Carlo Dionisi-Vici, Goknur Haliloglu, Daniela Karall, Kimberly A Chapman, Martina Huemer, Michel Hochuli, Murielle Assoun, Diana Ballhausen, Alberto Burlina, Brian Fowler, Sarah C Grünert, Stephanie Grünewald, Tomas Honzik, Begoña Merinero, Celia Pérez-Cerdá, Sabine Scholl-Bürgi, Flemming Skovby, Frits Wijburg, Anita MacDonald, Diego Martinelli, Jörn Oliver Sass, Vassili Valayannopoulos, Anupam Chakrapani

**Affiliations:** Division of Metabolism and Children’s Research Centre, University Children’s Hospital Zurich, Steinwiesstrasse 75, 8032 Zurich, Switzerland; University Children’s Hospital, Im Neuenheimer Feld 150, Heidelberg, 69120 Germany; Dietician, Hôpital Necker-Enfants Malades, 149, rue de Sèvres, Paris, 75015 France; Centre for Molecular Diseases, University Children’s Hospital, Avenue Pierre Decker 2, Lausanne, 1011 Switzerland; Department of Pediatrics, University of Padua, Via Giustiniani 3, Padova, 35128 Italy; Section of Genetics and Metabolism, Children’s National Health System, Washington DC, USA; Division of Metabolism, Bambino Gesù Children’s Research Hospital, Piazza S. Onofrio 4, Rome, 00165 Italy; Center for Pediatrics and Adolescent Medicine, University Medical Center Freiburg, Mathildenstr. 1, Freiburg, 79106 Germany; Metabolic Unit, Great Ormond Street Hospital, Institute of Child Health, Great Ormond Street, London, WC1N 3JH UK; Department of Pediatric Neurology, Hacettepe University Children’s Hospital, Ankara, Turkey; Department of Endocrinology, Diabetology and Clinical Nutrition, University Hospital Zurich, Zurich, Switzerland; Department of Pediatrics and Adolescent Medicine, First Faculty of Medicine, Charles University in Prague and General University Hospital in Prague, Prague, Czech Republic; Medical University Innsbruck, Clinic for Pediatrics I, Inherited Metabolic Disorders, Anichstrasse 35, Innsbruck, 6020 Austria; Dietetic Department, Birmingham Children’s Hospital, Steelhouse Lane, Birmingham, B4 6NH United Kingdom; Division of Metabolism, Bambino Gesù Children’s Hospital, IRCSS, Piazza S. Onofrio 4, Rome, I-00165 Italy; Centro de Diagnóstico de Enfermedades Moleculares, Facultad de Ciencias, Universidad Autónoma de Madrid, IDIPAZ, CIBER de Enfermedades Raras (CIBERER), Madrid, 28049 Spain; Division of Clinical Chemistry and Biochemisty, Children’s Research Centre, University Children’s Hospital Zurich, Steinwiesstrasse 75, 8032 Zurich, Switzerland; Department of Clinical Genetics, University Hospital, Rigshospitalet 4062, Blegdamsvej 9, Copenhagen, 2100 Denmark; Reference Center for Inherited Metabolic Disease and IMAGINE Institute, Hôpital Necker-Enfants Malades 149, rue de Sèvres, Paris, Cedex 15 75743 France; Department of Pediatrics, Academic Medical Center (AMC), Meibergdreef 9, Amsterdam, 1105 AZ the Netherlands; Department of Metabolic Medicine, Great Ormond Street Hospital, Great Ormond Street, London, WC1N 3JH UK

**Keywords:** Methylmalonic acidemia, Methylmalonic aciduria, Propionic acidemia, Propionic aciduria, Methylmalonyl-CoA mutase, Propionyl-CoA carboxylase, Vitamin B_12_/adenosylcobalamin, Biotin, Hyperammonemia, Metabolic decompensation, Metabolic stroke (-like event), Movement disorder, Seizures, Intellectual disability

## Abstract

Methylmalonic and propionic acidemia (MMA/PA) are inborn errors of metabolism characterized by accumulation of propionic acid and/or methylmalonic acid due to deficiency of methylmalonyl-CoA mutase (MUT) or propionyl-CoA carboxylase (PCC). MMA has an estimated incidence of ~ 1: 50,000 and PA of ~ 1:100’000 -150,000. Patients present either shortly after birth with acute deterioration, metabolic acidosis and hyperammonemia or later at any age with a more heterogeneous clinical picture, leading to early death or to severe neurological handicap in many survivors. Mental outcome tends to be worse in PA and late complications include chronic kidney disease almost exclusively in MMA and cardiomyopathy mainly in PA. Except for vitamin B_12_ responsive forms of MMA the outcome remains poor despite the existence of apparently effective therapy with a low protein diet and carnitine. This may be related to under recognition and delayed diagnosis due to nonspecific clinical presentation and insufficient awareness of health care professionals because of disease rarity.

These guidelines aim to provide a trans-European consensus to guide practitioners, set standards of care and to help to raise awareness. To achieve these goals, the guidelines were developed using the SIGN methodology by having professionals on MMA/PA across twelve European countries and the U.S. gather all the existing evidence, score it according to the SIGN evidence level system and make a series of conclusive statements supported by an associated level of evidence. Although the degree of evidence rarely exceeds level C (evidence from non-analytical studies like case reports and series), the guideline should provide a firm and critical basis to guide practice on both acute and chronic presentations, and to address diagnosis, management, monitoring, outcomes, and psychosocial and ethical issues. Furthermore, these guidelines highlight gaps in knowledge that must be filled by future research. We consider that these guidelines will help to harmonize practice, set common standards and spread good practices, with a positive impact on the outcomes of MMA/PA patients.

## Introduction

Methylmalonic and propionic acidemia (MMA/PA) are autosomal recessive disorders of propionate catabolism caused by defects in the enzymes methylmalonyl-CoA mutase (MUT) or propionyl-CoA carboxylase (PCC) characterized by accumulation of metabolites of branched-chain amino acid catabolism such as 3-hydroxypropionic acid, methylcitric acid and/or methylmalonic acid in plasma, urine and other body fluids.

**Figure 1:**
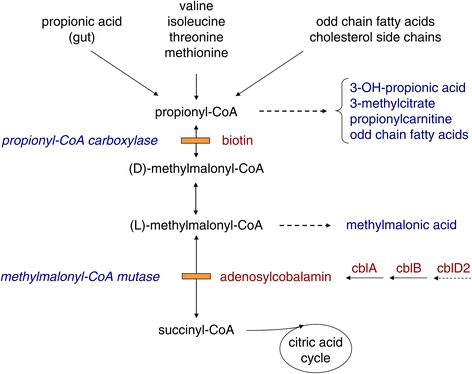
**Metabolic interrelationship of MMA and PA.**

Mitochondrial propionyl-CoA carboxylase (PCC, EC 6.4.1.3) is an α6β6 dodecamer composed of PCCA and PCCB subunits catalyzing the reversible biotin-dependent conversion of propionyl-CoA to D-methylmalonyl-CoA. This is racemised to its L-enantiomer, L-methylmalonyl-CoA which is reversibly isomerised to succinyl-CoA, catalyzed by L-methylmalonyl-CoA mutase (MUT, EC 5.4.99.2) which requires vitamin B_12_ (cobalamin) in the form of adenosylcobalamin (AdoCbl) as cofactor (Figure 1). These reactions represent crucial steps in propionate catabolism, funneling metabolites from the breakdown of the amino acids valine, isoleucine, methionine and threonine, odd-chain fatty acids and the side chain of cholesterol into the tricarboxylic acid cycle. While mutations in either the *PCCA* or *PCCB* gene cause propionic acidemia (MIM# 606054), isolated methylmalonic acidemia is caused either by a genetic defect in the MUT enzyme itself (MIM# 251000, MMA *mut* type), or in one of the proteins (MMAA, MMAB, MMADHC) involved in the synthesis of its active cofactor, adenosylobalamin (MMA *cblA* type, MIM# 251100; MMA *cblB* type, MIM# 251110; MMA *cblD-variant 2* MIM# 277410) [1]. The MUT apoenzyme deficiencies are subdivided into two subgroups, the mut° defect with virtually undetectable MUT activity and the mut^−^ defect with low to moderate residual MUT activity in the presence of high concentrations of AdoCbl. Defects in cobalamin metabolism may also manifest as combined methylmalonic aciduria and homocystinuria (*cblC, cblD, cblF* and *cblJ* defects) [2,3].

MMA and PA are rare disorders and the true incidence in Europe is unknown [4]. Estimates of incidence in Western populations range from 1:48,000 to 1:61,000 births for MMA [5] and from 1:50,000 to 1:500,000 births for PA. Overall incidence is believed to be ~ 1: 50,000 for isolated MMA and ~ 1:100’000 to 150,000 for PA [6]. In some populations across the world, the incidence is much higher. For example, PA incidence in Saudi Arabia is reported to be much higher at 1 in 2,000 to 5,000 live births [7].

Patients with a complete enzyme deficiency present in the first days to weeks of life with acute deterioration of their general clinical condition, metabolic acidosis and hyperammonemia, progressing to coma and death, if untreated. Late-onset cases of MMA and PA may present at any age, i.e. in infancy, childhood or even later with a more heterogeneous clinical picture. Mental outcome tends to be worse in PA and late complications include chronic kidney disease almost exclusively in MMA and cardiomyopathy mainly in PA. The overall outcome remains poor despite the existence of apparently effective therapy with a low protein diet and carnitine except for vitamin B_12_-responsive forms of MMA (mainly cblA type MMA), which have a better outcome if diagnosed timely and treated adequately. Since prognosis is strongly influenced by the duration of coma and peak blood ammonia concentrations, especially in neonates [8-10], patients must be identified and adequately treated as soon as possible. In view of the complexity of the resources required for rapid diagnosis, efficient timely management and intense monitoring of treatment, sufficient experience in the diagnosis and treatment (including extracorporeal detoxification) of inborn errors of metabolism (IEM) with supporting laboratory resources available 24 h/7 d is essential. However, the rarity of MMA/PA prevents single centers or even countries from having all the expertise for evidence-based management.

Currently, different guidelines for the diagnosis and treatment of MMA and PA are in place in some European institutions/countries, but there is no consensus on how to diagnose and treat patients with suspected or confirmed MMA/PA [11]. Therefore the aim of this consensus guideline is to standardize, systematize and harmonize the diagnosis, therapy and long-term management of MMA/PA in Europe based on the highest level of evidence, by pooling all the published evidence and experience of leading centers from several European countries and the U.S. These guidelines, developed using the SIGN methodology (Scottish Intercollegiate Guideline Network, http://www.sign.ac.uk), are intended for metabolic specialists, pediatricians, dietitians, neonatologists, intensive care specialists, adult physicians, obstetricians, nurses and psychologists involved in the care of MMA/PA patients.

## Methodology and objectives

### Guideline development

The process leading to this guideline was started at the annual symposium of the “Society for the Study of Inborn Errors of Metabolism” (SSIEM) held in Geneva in August 2011. Three further meetings were held in Zurich (March 2012), Birmingham (September 2012), and again in Zurich (January 2013). In the Geneva meeting the guideline development group (GDG) was trained on methodology to ensure standardized literature evaluation and working groups were established, focusing on specific guideline topics. Thereafter GDG members performed a systematic literature review, drafted the guideline, discussed it with all other GDG members in subsequent meetings, and also discussed the revisions of the guideline draft made by external consultants specialized on neonatology/intensive care (Jochen Meyburg, Heidelberg, child neurology (B. Plecko-Startinig, Zurich), nephrology (C.P. Schmitt, Heidelberg), and a patient group representative (S. Hannigan, London). Furthermore, revisions of the guideline were made by the GDG based on the judgments of two highly renowned external reviewers (M. Duran, Amsterdam, biochemist with specific expertise in organic acidurias; J. Walter, Manchester, experienced metabolic pediatrician, editor of the standard text book *Inborn Metabolic Diseases – Diagnosis and Treatment* (5th ed. 2012), Springer, Berlin).

The GDG consisted of pediatric metabolic specialists (D. Ballhausen, M. R. Baumgartner [chairman], A. B. Burlina, A. Chakrapani [co-chairman], K. Chapman [representing PA guideline group from USA], C. Dionisi-Vici, S. C. Grünert, S. Grünewald, F. Hörster [secretary], T. Honzik, D. Karall, S. Scholl-Bürgi, F. Skovby, V. Valayannopoulos, F. Wijburg), biochemical geneticists/clinical biochemists (B. Fowler, B. Merinero, C. Pérez-Cerdá, J. O. Sass), specialist metabolic dieticians (A. MacDonald, M. Assoun, S. Dubois [Paris], E. Müller, Heidelberg), pediatric neurologists (G. Haliloglu, D. Martinelli), a psychologist/metabolic pediatrician (M. Huemer), and an adult metabolic specialist (M. Hochuli). The guideline group meetings were supervised by a moderator (M. Summar, Washington) whose role was to oversee the discussion without directly contributing to the content of the guideline. The practical applicability of this guideline has been pilot-tested and supported by 3 pediatricians in training (P. Forny, A. Lämmle, A. Schumann) who were asked to read it and to provide comments. The final guideline will be sent to all European societies for inborn errors of metabolism for endorsement.

### Systematic literature review and evidence grading

The methodology used for collecting the evidence base for this guideline is essentially that used by the Scottish Intercollegiate Guideline Network (SIGN, http://www.sign.ac.uk). A systematic literature review on MMA/PA from the time of description of each disease until December 2011 was carried out using mainly Medline, Embase, the Cochrane Library, MedLink, and Orphanet. A few papers which were published later and were considered by the group as important were included after that time point. Searches also included websites of international and national societies and parent groups for inborn errors. Relevant papers were evaluated by a minimum of two members of the GDG before conclusions were considered as evidence.

Evidence levels were classified in accordance with the SIGN methodology (Table 1) and recommendations given in the guideline were graded depending on their level of evidence (Table 2).

**Table 1:** **Evidence levels were classified in accordance with the SIGN methodology**

**Evidence level**	**Criteria**
1^++^	High quality meta-analyses, systematic reviews of randomized control trials (RCTs), or RCTs with a very low risk of bias.
1^+^	Well conducted meta-analyses, systematic reviews of RCTs, or RCTs with a low risk of bias.
1^−^	Meta-analyses, systematic reviews or RCTs, or RCTs with a high risk of bias.
2^++^	High quality systematic reviews of case-control or cohort studies or high quality case-control or cohort studies with a very low risk of confounding bias, or chance and a high probability that the relationship is causal.
2^+^	Well conducted case-control or cohort studies with a low risk of confounding, bias, or chance and a moderate probability that the relationship is causal.
2^−^	Case-control or cohort studies with a high risk of confounding, bias, or chance and a significant risk that the relationship is not causal.
3	Non-analytic studies, e.g. case reports, case series.
4	Expert opinion.

**Table 2:** **Grading of recommendations depending on their level of evidence**

**Grade of recommendation**	**Criteria**
A	If level 1 evidence was found (not the case in this study).
B	If level 2 evidence was found.
C	If level 3 evidence was found (mainly non-analytical studies such as case reports and case series).
D	If level 4 evidence was found (mainly expert opinion).

### Disclaimer

These guidelines are intended to help decision making in MMA/PA patient care. Although based on the best available evidence, the consensus recommendations often only represent expert opinion and are meant to be followed flexibly applying own experience and considering the individual patient. Guidelines cannot guarantee satisfactory diagnosis and outcome in every patient. Furthermore, although as exhaustive as possible, these guidelines cannot include all possible methods of diagnostic work-up and care and may inadvertently omit some acceptable and established procedures. Although they should help to optimize the care of individual patients and assist decision making by basing clinical practice on scientific and medical knowledge the guidelines should not substitute well-informed, prudent clinical practice.

### Diagnosis (and differential diagnosis)

#### Clinical signs and symptoms - conditions raising the suspicion of MMA/PA

Clinical signs and symptoms of MMA/PA are nonspecific. Patients may present with acute or chronic symptoms at any age (Table 3). Some of the signs and symptoms are common, others are uncommon and a few are only described in single cases.

**Table 3:** **Acute and chronic presentations of MMA/PA**

**Acute presentation**	**Chronic presentation**
**Neonatal sepsis-like picture, temperature instability, respiratory distress, hyperventilation**	**Often episodic characteristic signs and symptoms**
**Nervous system**	**Nervous system**
**• Altered level of consciousness (from lethargy and somnolence to coma) mimicking encephalitis or drug intoxication**	**•** Hypotonia
	**• Developmental delay** (learning disabilities, intellectual disability)
**•** Acute encephalopathy	**• Movement disorders/dystonia**
**•** Seizures (in general not isolated but in the context of altered level of consciousness)	**•** Seizures
**•** Movement disorders (more frequent in PA)	**•** Optic atrophy
**•** Stroke-like episodes (more frequent in MMA)	**•** *Psychiatric symptoms (hallucinations, psychotic attacks)*
**Gastrointestinal system**	**Gastrointestinal system**
**• Vomiting and feeding difficulties**	**• Recurrent vomiting with ketoacidosis**
	**•** Abnormal feeding behavior (anorexia)
	**• Failure to thrive**
	**•** Constipation
	**•** Pancreatitis
**Hematologic findings**	**Hematologic findings**
**•** Neutropenia, pancytopenia	**•** Neutropenia, pancytopenia
	**•** *Secondary hemophagocytosis (rare)*
**Heart**	**Heart (more frequent in PA)**
**•** Acute cardiac failure (mostly on basis of cardiomyopathy)	**•** Cardiomyopathy
**•** Arrhythmias	**•** Prolonged QTc interval in ECG
	**Kidney (more frequent in MMA)**
	**• Chronic renal failure in MMA**
	**Other**
	**•** *Dermatitis*
	**•** *Hearing loss*

**bold**: typical signs and symptoms.

standard: uncommon signs and symptoms.

*italics*: signs and symptoms only reported in single patients.

*Grade of recommendation: D.*

In the classical, neonatal onset form of MMA/PA, symptoms start as early as the second day of life with acute deterioration of the general clinical condition, vomiting, dehydration, weight loss, temperature instability, neurological involvement with muscular hypo- or hypertonia, irritability, lethargy progressing to coma and seizures (Table 3). At presentation, laboratory findings include severe and persistent metabolic acidosis and ketosis, elevated anion gap and hyperammonemia. As in any sick newborn, sepsis and other more common conditions such as birth trauma, gastrointestinal obstruction, and cardiorespiratory difficulties should be excluded [7,12,13].

##### Statement #1: Grade of recommendation C

In newborns with clinical distress and/or suspicion of sepsis organic acidemias must be considered in the differential diagnosis from the outset (see Tables 3 and 4 and section on laboratory diagnosis).

**Table 4:** **Bedside differential diagnostics of inborn errors of metabolism presenting with acute encephalopathy (modified from Haeberle et al.** [14]**)**

**Parameter**	**Condition**								
	**UCD**	**MMA/PA**	**β-Keto-thiolase deficiency**	**MSUD**	**β-oxidation defects**	**HMG CoA lyase deficiency**	**HIHA**	**Mitochondrial/PC deficiency** ^**e**^	**PDH deficiency**
↑ NH_3_	++	+	–	–	+/–	+	+	+/–	–
Acidosis	+/–	+	++	–	+/–	+	–	+	+
Ketonuria^a^	–	++/+++	+++	+/++	–	–	–	+/++	–
Hypoglycemia^b^	–	+/–	–	–	+	+	++	+/–	–
↑ Lactic acid^c^	–	+	+	–	+/–	+	–	++	++
↑ AST & ALT	(+)	+/–	–	–	++	+/–	–	+/–	–
↑ CPK	–	–	–	–	++	–	–	+/–	–
↑ Uric acid	–	+	+	+	+	+	–	+/–	+/–
↓ WBC/RBC/Plt	–	+	–	–	–	–	–	+/–	–
Weight loss	–	+^d^	+	+/–	–	–	–	+	–

Non-standard abbreviations include: MSUD, maple syrup urine disease; HMG-CoA lyase, 3-hydroxy-3-methlyglutaryl-CoA lyase; HIHA, Hyperinsulinism-hyperammonemia syndrome; PC, pyruvate carboxylase; PDH, pyruvate dehydrogenase.

^a^Ketonuria (++ - +++) suggests OA in neonates.

^b^Hypoglycemia and hyperammonemia (“pseudo-Reye”) are predominant in 3-HMG-CoA-lyase deficiency (more than in PC deficiency).

^c^Lactic acid elevation refers to a plasma lactate > 6 mmol/l; lower levels of 2-6 mM may be due to violent crying or extensive muscle activity.

^d^Only in neonates.

^e^Only type B associated with hyperammonemia but not types A and C.

*Grade of recommendation: D.*

After the neonatal period, symptoms of MMA/PA may vary considerably and affect different organ systems such as the nervous system, gastrointestinal tract, immune system, heart (mainly in PA) and kidney (mainly in MMA). Importantly, metabolic crises are frequently triggered by catabolic events, protein overload or certain drugs. Symptoms may also mimic other more common conditions such as diabetic ketoacidosis with hyperglycemia [15-19] or Reye syndrome [20]. Common nonspecific symptoms/conditions include encephalopathy or unexplained coma [21-23], failure to thrive [21-33], muscular hypotonia [21-23,27,30,34], epilepsy [13,23-28,35], neuropsychiatric symptoms [36,37], cardiomyopathy and prolonged QTc interval (the latter only in PA) [21,38-45], and progressive renal insufficiency [25,26,46-54].

##### Statement #2: Grade of recommendation C

After the neonatal period, the clinical presentation of MMA/PA may mimic other more common conditions. Affected systems are (see Table 3):

- Gastrointestinal tract: recurrent vomiting with ketoacidosis, abnormal feeding behavior, failure to thrive, constipation, pancreatitis.
- Nervous system: acute encephalopathy, hypotonia, seizures, developmental delay, movement disorders/stroke-like events, psychiatric symptoms.
- Hematologic findings: neutropenia, involvement of bone marrow.
- Heart: cardiomyopathy, prolonged QTc interval (mainly in PA).
- Kidney: chronic renal failure in MMA.

Published reports on the natural history of MMA/PA do not usually differentiate between clinical presentation leading to the diagnosis and symptoms appearing during the course of the disease. The frequency of different signs and symptoms are listed in Table 5. It must be noted that some rare symptoms may be over represented and some common symptoms under-represented in the literature implying a significant publication bias.

**Table 5:** **Frequencies of signs & symptoms reported in MMA/PA**

**Sign/symptom**	**Frequency**		**References**	
	**MMA**	**PA**	**MMA**	**PA**
**Developmental delay**	25-65%	59-100%	[25,26,29]	[21-24,27,30,32,33,55]
**Encephalopathy**	Frequent during metabolic crises, but no specific data	21-30%	[26]	[7,21-23,56,57]
**Hypotonia**	No data	56-100%		[21-23,27,30]
**Seizures/epilepsy**	16-53%	25-53%	[25,26,58]	[7,13,23,24,27,35,55,59]
**Movement disorder**	30-45%	40%	[25,29,58]	[33]
**Metabolic stroke like events & basal ganglia lesions**	Up to 35%	>10 cases	[26,29,48,58]	[7,20-22,35,55,59-65]
**Optic atrophy**	Single cases	>10 cases	[66-68]	[22,68,69]
**Neuropsychiatric symptoms**	Rare	rare	[25]	[36,37]
**Chronic renal failure**	28-47%	4 cases	[25,26,48]	[27,55,70]
**Cardiomyopathy**	Few cases	9-23%	[26,71]	[32,44,55]
**Prolonged QTc interval**	Not reported	37 cases		[21,22,31,38,40,41,55,56]
**Pancreatitis**	22 cases	7 cases	[25,26,72,73]	[21,22,72,74-76]
**Immunodeficiency**	Rare	rare	[77]	[7,77-79]

##### Statement #3: Grade of recommendation C

Rare(r) clinical symptoms or manifestations of MMA and PA as a single (presenting) symptom or in combination with other symptoms have been described. They should be considered in the diagnosis of MMA/PA and must be considered in the follow-up and monitoring of previously diagnosed patients.

A careful medical and family history is mandatory and should include questions about unexplained neonatal deaths or neurological disorders in the family, consanguinity, evidence of protein avoidance in the patient and siblings, and drug intake by the patient.

### Laboratory findings

#### Baseline laboratory tests raising suspicion of MMA or PA

MMA and PA should be considered in any newborn/child (whether critically ill or not) with unexplained

- Metabolic acidosis (with elevated anion gap)
- Elevated lactate
- Hyperammonemia
- Leukopenia, thrombocytopenia, anemia [27] and/or
- Urine ketone bodies (acetoacetate, dipstick) [80]

##### Statement #4: Grade of recommendation C

Metabolic acidosis (with elevated anion gap), elevated lactate, hyperammonemia, elevated urinary ketone bodies (in particular in newborns) are laboratory hallmarks of MMA and PA and therefore should be investigated in any critically ill patient or unexplained condition.

If hyperammonemia is present, determination of plasma amino acids, blood or plasma acylcarnitines and urinary organic acids and orotic acid should be urgently requested together with basic laboratory investigations. Treatment must be commenced immediately on presentation without waiting for these results, which must be available within 24 hours. When samples are taken after recovery from an acute episode, urinary organic acids may be especially helpful for diagnosis. In patients with a fatal outcome, a skin biopsy is recommended for the establishment of cultured fibroblasts, along with anticoagulated blood for DNA isolation/immortalization of lymphocytes and stored frozen aliquots of plasma, serum and urine [81].

##### Statement #5: Grade of recommendation D

If ammonia is increased, further metabolic investigations should be performed immediately but specific treatment must not be delayed.

#### Differential diagnosis

The most common misdiagnosis of neonatal onset MMA/PA is sepsis. Standard clinical and analytical procedures generally differentiate between hyperammonemia due to inborn errors and that due to other conditions such as liver failure. Table 4 lists inborn errors of metabolism leading to acute deterioration with encephalopathy and hyperammonemia guiding bedside differential diagnosis (for further reading see also [14]). Metabolic acidosis, elevation of lactate and anion gap, and disturbances of glucose metabolism may help to differentiate MMA/PA from other disorders presenting with acute deterioration and encephalopathy. Lack of megaloblastic anemia with increased MCV and elevated plasma homocysteine differentiates from vitamin B_12_ deficiency and disorders of intracellular cobalamin metabolism.

#### Specialized biochemical investigations

Subsequent investigations include determination of organic acids in urine, amino acids in blood (plasma, drawn ideally after 3-4 hours fasting), urine and CSF, acylcarnitine profile in blood (dried blood or plasma) and total plasma homocysteine which is essential in order to differentiate the various types of MMA [1]. Methylmalonic acid is not elevated in PA and thus allows distinction between MMA and PA [1] using urinary organic acid analysis. While methylcitrate and 3-hydroxypropionic acid are present in both disorders, N-propionylglycine, N-tiglylglycine, 2-methyl-3-oxovaleric acid, 3-hydroxy-2-methylbutyric acid, 2-methyl-3-oxobutyric acid, 3-hydroxy-n-valeric acid, 3-oxo-n-valeric acid, are detectable in PA only [18,27]. In the acylcarnitine profile propionylcarnitine (C3) is elevated [34,82], but this is not specific and does not help to differentiate between MMA and PA [34]; elevated methylmalonylcarnitine (an isomer of C4DC) can be found in MMA [83,84]. Amino acid analysis usually shows elevated glycine and lysine concentrations in blood, urine [27] and CSF, but normal glycine CSF/plasma ratio [35]. Determination of odd numbered long-chain fatty acids (OLCFA) in erythrocyte membranes [85,86] or plasma [87,88] is an additional diagnostic aid but is not routinely used and only performed in few laboratories.

##### Statement #6: Grade of recommendation B-C

Determination of organic acids in urine and the acylcarnitine profile in blood are the most commonly used investigations to detect MMA and PA. Determination of amino acid concentrations may help in diagnosis and treatment. In addition total plasma homocysteine allows differentiation between the various types of MMA.

#### Confirmation of diagnosis by enzyme and molecular genetic investigations

The complexity of inherited isolated MMA/PA usually requires characterization of the underlying defect in cultured skin fibroblasts and/or lymphocytes and/or molecular genetic analysis. A favored approach is initial measurement of the overall conversion of propionate to succinate by the incorporation of label from [^14^C] propionate into cell proteins in normal and vitamin B_12_ supplemented medium [89]. For MMA the ratio of activity in B_12_-supplemented versus unsupplemented medium can allow distinction of mut° and some cases of cblB (ratio below 1.5) from mut^−^ and cblA (ratio higher than 1.5) [90]. Measurement of specific activity of MMCoA mutase in the presence and absence of Ado-Cbl [91] is needed in cases that remain unclear. This approach allows selection of the appropriate gene for mutation analysis. In some cases such as a known mutation in a family member or common mutation in a particular population this approach may be rationalized by early or direct performance of mutation analysis.

For PA, assay of PCC in lymphocytes and/or cultured fibroblasts is the more reliable and rapid (in lymphocytes) method to confirm the disease. Either complementation or mutation analysis confirms the genetic defect which is essential to offer prenatal diagnosis to the families.

##### Statement #7: Grade of recommendation B/D

Enzymatic studies and/or molecular genetic analyses should be performed to confirm diagnosis (B). This is ideally done in specialized laboratories (D).

For MMA, knowledge of the underlying enzymatic defect [25,92] and the underlying genotype (mut^0^, mut^−^, cblA, cblB or cblD-variant 2) [1,87,90,93-95] is of great importance since residual enzyme activity and vitamin B_12_-reponsiveness influences the clinical course and can be associated with a better long term outcome.

The spectrum of genetic defects and the range of causative mutations are different in different populations of MMA [90,95-97] and PA patients [32,94,97-102]. A few common mutations are found in each defect; however, most mutations are private. The identification of disease-causing mutations facilitates accurate prenatal diagnosis, determination of carrier status for family members, genetic counseling and in some cases genotype-phenotype correlations [48,90,94,95,103].

##### Statement #8: Grade of recommendation C-D

Defects in different genes can cause isolated methylmalonic aciduria. The clinical phenotype is influenced by the underlying enzymatic defect (mut^0^, mut^−^, cblB, cblA and cblD-variant 2) and genotype (*mut*, *MMAA*, *MMAB, MMADHC*).

##### Statement #9: Grade of recommendation B

No clear-cut genotype-phenotype correlations have been found in PA.

#### Prenatal testing

Prenatal testing in both diseases is feasible. Prior to testing, it is desirable that the index case has been confirmed biochemically and/or genetically, and the carrier status of the parents has been confirmed by mutation analysis [104,105]. Mutation analysis in fetal DNA is the method of choice. To improve reliability or if mutation analysis of the index case is not available additional tests may be carried out including determination of metabolites in amniotic fluid (methylcitrate and/or propionylcarnitine in PA; methylmalonic acid/methylcitrate in amniotic fluid or amniotic fluid dried on filter paper in MMA) [106] or activity assay in amniocytes or intact native or cultured chorion villi. Preimplantation genetic diagnosis (PGD) has been reported as a reproductive option for couples affected with PA [107].

##### Statement #10: Grade of recommendation D

Prenatal testing in both diseases is feasible. Prior to testing, it is desirable that the index case has been confirmed biochemically and/or genetically, and the carrier status of the parents has been confirmed by mutation analysis.

#### Newborn screening (NBS)

Newborn screening for MMA and PA is technically feasible using propionylcarnitine and methionine and has been implemented in some countries (e.g. Austria, U.S, Spain, Italy), but not in others (e.g., Germany, France, U.K, Netherlands). Because both markers lack disease specificity, several attempts using analyte ratios and 2^nd^ tier testing have been undertaken to differentiate between false and true positives [34,108-110] but so far yielded conflicting results in patient studies [111,112]. Promising results regarding false-positive rate and positive predictive value were obtained in a study using a 2^nd^ tier method for the detection of total homocysteine, methylmalonic acid and methylcitrate [113].

The most comprehensive study investigated 55 PA patients 20 of which were diagnosed via newborn screening [21]. 63% of the newborn screening patients were already symptomatic at the time of diagnosis. The authors conclude that early diagnosis of PA through newborn screening seems to be associated with a lower mortality rate. However, no significant benefit could be shown for surviving patients with regard to their clinical course, including the number of metabolic crises, physical and neurocognitive development, and long-term complications [21,22].

##### Statement #11: Grade of recommendation C-D

Newborn screening for MMA and PA is technically feasible. So far available data about outcome has not answered the question as to whether newborn screening in MMA/PA is of long-term clinical benefit.

### Acute management

#### Initial management

Since the long-term neurodevelopmental outcome is strongly influenced by the duration of coma and peak blood ammonia concentrations [114-116], therapy must not be delayed and therefore the diagnostic workup and the initial medical treatment should proceed simultaneously:

1. Stabilize the patient.
2. Stop protein intake.
3. Start intravenous glucose.
4. Seek expert metabolic advice.
5. Initiate first-line treatment as outlined in Table 6.
6. Collect samples (blood spot, plasma and urine) for diagnostic purposes.

**Table 6:** **Management of symptomatic hyperammonemia in undiagnosed patients and known patients with MMA/PA**

**Ammonia level (μmol/l)**	**Action in undiagnosed patient**	**Action in known MMA/PA patient**	**Comments**
Increased > upper limit of normal	**•** Stop protein intake	**•** Stop protein intake	**•** Stop protein for maximal 24 (-48) hours
	**•** Give iv glucose at appropriate dosage (see Table 7) to stop catabolism ± insulin^#^	**•** Give iv glucose at appropriate dosage (see Table 7) to stop catabolism ± insulin	
			**•** Avoid exchange transfusions as cause of catabolism/protein load
	**•** Monitor ammonia blood levels every 3 hours	**•** Increase carnitine dosage to 200 mg/kg/d	
			**•** Hyperglycemia can be extremely dangerous (hyperosmolarity)
		**•** Monitor ammonia blood levels every 3 hours	
100-250*	**•** As above	**•** As above	
	**•** Start drug treatment with i.v. arginine, sodium benzoate and sodium phenylbutyrate (see Table 7)	**•** Start drug treatment with sodium benzoate (see Table 7)	
			**•** If major hyperglycemia occurs with increasing lactate reduce glucose infusion rather than increasing insulin
	**•** Start carbamylglutamate, carnitine, vitamin B_12_ (preferably hydroxo-Cbl), and biotin (see Table 7)	**•** Consider carbamylglutamate (see Table 7)	
			**•** Avoid hypotonic solutions
250-500	**•** As above	**•** As above	**•** Add sodium and potassium according to the electrolyte results (cave hypokalemia when acidosis is corrected)
	**•** Prepare extracorporeal detoxification if significant encephalopathy and/or early high blood ammonia level or very early onset of disease (day 1 or 2)	**•** Consider extracorporeal detoxification dependent on patient’s age and history	
			**•** Take into account the sodium intake if sodium benzoate or phenylbutyrate is used^§^
	**•** Begin extracorporeal detoxification if no rapid drop of ammonia within 3-6 hours		
			**•** Avoid repetitive drug boluses
500-1000	**•** As above	**•** As above	**•** Monitor phosphate levels and supplement early esp. during hemodialysis
	**•** Start extracorporeal detoxification immediately		
>1000	**•** Evaluate whether to continue specific treatment or to start palliative care	**•** As above	

^*^This limit of action applies for patients outside the neonatal period; for neonates use >150 and <250.

^#^Monitor blood glucose after 30 min and subsequently every hour, because some neonates are very sensitive to insulin.

^§^1 g sodium benzoate and sodium phenyl butyrate contain 7 mmol Na and 5.4 mmol Na, respectively.

*Grade of recommendation: C-D.*

While waiting for the laboratory diagnosis, treatment should be started without delay with medications as outlined in Table 7.

**Table 7:** **Dosage of drugs in acute hyperammonemia**

**Glucose IV**	**L-carnitine IV**	**Hydroxo-cobalamin# IV/IM**	**Biotin IV/PO**	Sodium benzoate* (to be given IV in glucose 10%)	°Sodium phenylbutyrate* (to be given IV in glucose 10%)	^**§**^ L-arginine-HCl* (to be given IV in glucose 10%)	**N-carbamyl-glutamate PO**
Age dependent (see Table 8)	100 mg/kg as bolus, then maintenance 100 mg/kg/d	1 mg/day	10 - 40 mg/day	250 mg/kg as bolus in 90-120 min, then maintenance dose 250 mg/kg/d	250 mg/kg as bolus in 90-120 min, then maintenance dose 250 mg/kg/d	250 mg/kg as bolus in 90-120 min, then maintenance dose 250 mg/kg/d	100 mg/kg bolus, then 25-62 mg/kg every 6 h

#Vitamin B_12_ is preferably given in the form of hydroxocobalamin; cyanocobalamin is less efficient but may be used temporarily.

*Maximal daily drug dosages: sodium benzoate 5, 5 g/m^2^or 12 g/d, sodium PBA 5, 5 g/m^2^or 12 g/d, L-arginine 12 g/day.

°Sodium phenylbutyrate should only be used in urea cycle defects or when the cause of hyperammonemia is unknown. In severe acute decompensation both sodium benzoate and sodium PBA/phenylacetate should be given in parallel as “ultima ratio”. In less severe cases, a stepwise approach with initial sodium benzoate and if hyperammonemia persists or worsens, the addition of sodium PBA/phenylacetate can be chosen.

^§^Arginine should only be used when the cause of hyperammonemia is unknown or when plasma arginine is low.

*Grade of recommendation: D.*

##### Statement #12: Grade of recommendation C-D

One of the most severe life threatening events in MMA and PA is hyperammonemia. The acute management differs depending on whether the cause of hyperammonemia is known or not. The differential diagnosis should include urea cycle defects and some other inherited disorders (see Table 4). The start of ammonia detoxification and measures to reverse catabolism must not be delayed.

#### Medications and rationale in acute hyperammonemia

Table 7 gives an overview of drugs to be administered in a patient with acute hyperammonemia. It reflects the consensus of this guideline working group and is supported by several publications (see Häberle et al. (2012) for review [14]).

L-carnitine is given to compensate for secondary carnitine deficiency caused by urinary loss of carnitine-bound to organic acids. L-carnitine therapy is considered safe.

Ammonia scavengers are drugs that allow bypassing of the urea cycle, by conjugation of benzoate with glycine to generate hippurate, and of phenylacetate (phenylbutyrate is the precursor of phenylacetate) with glutamine to generate phenylacetylglutamine. The use of ammonia scavengers, which represents the mainstay of therapy for detoxification of ammonia in urea cycle defects, is still debated in MMA and PA as there is the theoretical risk of increasing intramitochondrial accumulation of CoA esters and of further depleting free CoA availability [4,117-119]. However, sodium benzoate has been reported to be safe and efficacious to treat hyperammonemia [120] and many metabolic centers regularly use this drug in organic acidurias. The use of sodium phenylbutyrate in MMA and PA raises further concern because in these diseases hyperammonemia is usually associated with decreased levels of glutamine, because the mechanism producing hyperammonemia differs from urea cycle defects. Due to the risk of further depletion of the glutamine/glutamate pool, the routine use of sodium phenylbutyrate or phenylacetate to treat hyperammonemia should be considered with extreme caution in MMA and PA [117-119]. Once the diagnosis of organic acidemia is established, phenylbutyrate/acetate should be discontinued [9].

Arginine administration aims at maximizing ammonia excretion through the urea cycle. After the diagnosis of MMA or PA is confirmed L-arginine treatment can be discontinued but arginine levels should still be monitored.

Vitamin B12 responsiveness should be systematically tested but is more likely in late-onset MMA forms than in patients presenting in the newborn period. Vitamin B12, the cofactor precursor of methylmalonyl-CoA mutase, should be tried in all suspected cases by giving 1mg hydroxocobalamin i.m. (for more details see [1]; cyanocobalamin is less efficient but may be used temporarily until OHCbl is available). Biotin is the treatment of choice in both holocarboxylase synthetase (HCS) and biotinidase deficiency while there are doubts whether biotin-responsive forms of PA truly exist.

N-Carbamylglutamate is an analogue of N-acetylglutamate that allosterically activates carbamylphosphate synthetase I in the urea cycle. This drug has been utilized in MMA and PA for its ability to antagonize propionyl-CoA induced hyperammonemia. The dose selection of N-carbamylglutamate, which is so far only available as an oral medication, differs in the literature [4,9,10,16,121-128]; we recommend to use the doses as suggested by Häberle et al. (2012) [14].

##### Statement #13: Grade of recommendation C-D

Initial management includes stopping protein intake and starting intravenous glucose. Combined treatment including parenteral L-carnitine, hydroxocobalamin, sodium benzoate, L-arginine, and oral biotin and N-carbamylglutamate should be given while waiting for the laboratory diagnosis. In diagnosed PA patients the continuation of biotin treatment is questionable because there are doubts whether biotin-responsive forms of PA truly exist.

#### Promotion of anabolism

The aim is to prevent endogenous catabolism, in particular protein catabolism, whilst providing enough energy to meet metabolic demands [129]. Intravenous fluids containing 10% glucose or higher concentration should be infused according to the patient’s age (Table 8). Insulin can be carefully used to promote anabolism while maintaining normoglycemia [4,9,130]. Patients on insulin and high glucose infusion should be monitored for increases of lactic acid due to a potential interference with Krebs cycle entry and inhibition of pyruvate dehydrogenase by toxic metabolites [127]. The dose of insulin, starting from 0.01-0.02 units/kg/h, must be adjusted frequently in order to control glycemia. Sustained normalization of blood glucose levels, which is an indirect marker of effective anabolism, allows insulin withdrawal. Caution in the use of insulin is recommended when lactic acidosis is present (plasma lactate >5 mmol/L). Lipid emulsion should be commenced early to provide additional calories at a dose of up to 2 g/kg/day. Platelets and triglycerides should be monitored during lipid treatment.

**Table 8:** **Age-dependent glucose requirement (mg/kg/min)**

**0-12 months**	**1-3 years**	**4-6 years**	**7-12 years**	**adolescent**	**adults**
8-10	7-8	6-7	5-6	4-5	3-4

Following improvement of metabolic and clinical abnormalities, natural protein preferably, should be reintroduced rapidly with the aim of meeting safe levels of protein intake (FAO/WHO/UNU 2007) and not withheld more than 24(-48) hours. Enteral feeding should be started as soon as the clinical condition allows.

##### Statement #14: Grade of recommendation C-D

To reverse endogenous catabolism, in particular protein catabolism, it is necessary to provide enough energy to meet the metabolic demands. Intravenous fluids containing glucose should be infused and insulin may be used to promote anabolism; after exclusion of a fatty acid oxidation disorder lipid emulsion should be commenced early to provide additional calories. Following improvement of metabolic and clinical abnormalities, protein should be rapidly reintroduced. Enteral feeding should be started as soon as possible.

#### Parenteral nutrition

Total parenteral nutrition (TPN) is the method of choice in infants with severe illness. An amino acid free parenteral solution is suitable for the first 24-48 h but protein must then be added using commercially available standard amino acid-solutions (containing essential and non-esential amino acids) [117]. NaCl and KCl should be progressively decreased to 2 g/l and 1.5 g/l, respectively [117]. Initially, amino acids are introduced in an amount sufficient to meet the age dependent safe levels of protein intake (FAO/WHO/UNU 2007), and then titrated according to biochemical monitoring of amino acids (see section on metabolic follow-up and monitoring). The minimal isoleucine requirement in neonates is at least equal to that of valine, but many i.v. amino acid solutions provide less of the former than the latter. Consequently, when the TPN solution only provides the minimal requirement for L-valine, additional oral supplementation of L-isoleucine (25–100 mg/day) is often necessary. Vitamins, minerals and micronutrients must always be provided to prevent selective deficiencies.

##### Statement #15: Grade of recommendation D

Parenteral nutrition is indicated, when enteral feeding cannot be established within 24-48 h. Amino acids are gradually introduced to meet safe levels of protein intake (see section on dietary treatment). An additional oral supplementation of L-isoleucine is often necessary. Vitamins, minerals and micronutrients must always be provided to prevent selective deficiencies.

#### Extracorporeal detoxification

Extracorporeal detoxification should be started in neonates and children who have blood ammonia levels >400-500 μmol/l, or if there is an inadequate response to medical therapy after 3-6 hours (this is the estimated time needed for preparing dialysis, including vascular access [8-10]). In late childhood or in adults, given the high susceptibility to developing severe brain edema, dialysis should be started earlier, i.e. if ammonia exceeds 200 μmol/l [14].

##### Statement #16: Grade of recommendation C-D

Extracorporeal detoxification is commonly used in severely decompensated patients. Persistent hyperammonemia, metabolic acidosis and severe electrolyte imbalances are indications for extracorporeal detoxification. Extracorporeal detoxification should be considered and preparation started in neonates and children who have blood ammonia levels >400-500 μmol/l. In late childhood or in adults dialysis should be considered even at lower ammonia levels.

The method of choice for extracorporeal detoxification in neonates and infants is continuous veno-venous hemodiafiltration (CVVHDF) [8,10]. In adults, either CVVHDF or hemodialysis (HD) are recommended. CVVHDF is a continuous procedure with excellent ammonia clearance and is usually well tolerated in infants. HD is an intermittent technique and provides the highest ammonia extraction, but its use in infants can cause severe technical and hemodynamic complications. In centers with less experience in extracorporeal detoxification, peritoneal dialysis can be utilized as first line intervention. However, its use is less effective in the acute setting compared with CVVHDF and HD [8].

The start of extracorporeal detoxification must not be delayed unless a decision for withdrawal of treatment and for palliative care is made.

##### Statement #17: Grade of recommendation C-D

The method of choice for extracorporeal detoxification in neonates and infants is continuous veno-venous hemodiafiltration (CVVHDF). In adults, both CVVHDF and hemodialysis (HD) are recommended. The mode of dialysis should be adjusted to the local experience and facilities. Whenever possible patients should be transferred to qualified centers.

#### General critical care management

The severity of metabolic decompensation does not depend on hyperammonemia alone but also other factors have to be taken into account, monitored and treated accordingly. Neonates and infants with organic acidurias and severe ketoacidosis present with intracellular dehydration that is often underestimated. In this situation, aggressive rehydration with hypotonic fluids and alkalization may cause or exacerbate pre-existing cerebral edema. Therefore, rehydration should be planned over a 48-h period, with a fluid infusion of about 150 ml/kg/24 h [117]. Acidosis can be cautiously corrected with i.v. bicarbonate, especially if it does not improve with the first measures of toxin removal. However, aggressive therapy with repeated boluses of i.v. bicarbonate may induce hypernatremia, cerebral edema, and even cerebral hemorrhage. Proposal of a precise dosage and timing of bicarbonate therapy is inappropriate since patients in metabolic crisis often present with severe blood electrolyte and osmolar abnormalities whose correction is managed by intensive care physicians. Furthermore, there are no criteria which define the degree of decompensation by clinical and laboratory parameters as severe, moderate, or mild in an organic aciduria. The severity of acidosis (pH ≤ 7.1) and level of ammonia (≥400/500 μmol/L) have been considered as the main discriminating variables, but other laboratory values (e.g. blood glucose, electrolyte and trace element levels, blood osmolarity, liver and kidney function, etc.) or clinical findings (e.g. degree of dehydration, cardiac and hemodynamic status, presence of pancreatitis, severity of the neurological picture) may contribute to an overall assessment of the level of severity. The supportive measures are applied in parallel to the procedure for toxin removal that, in addition to the dialysis of the toxic organic acids, can compensate for some of the fluid and electrolyte imbalance and allow for nutritional support.

##### Statement #18: Grade of recommendation D

Neonates and infants with organic acidurias and severe ketoacidosis present with intracellular dehydration that is often underestimated. Therefore adequate rehydration is essential. However, over aggressive hydration and alkalinisation may cause or exacerbate cerebral edema.

#### Additional treatments (under metabolic expert guidance)

In MMA, forced diuresis and alkalinisation of urine with sodium bicarbonate may help to eliminate methylmalonic acid due to its high renal clearance.

Glutathione deficiency and oxoprolinuria have been reported in a decompensated patient with MMA [131] and in another case high-dose ascorbate therapy (120 mg/kg/day) was effective in reducing lactic acidosis and oxoprolinuria [132].

Metabolic decompensation may be complicated by severe lactic acidosis due to thiamine deficiency, requiring vitamin supplementation.

No data exist in literature about neuroprotection in acute management in MMA/PA patients. Hypothermia, anti-inflammatory agents and NMDA receptor blockers are now routinely used as a neuroprotective strategy in several emergency conditions and their use in MMA and PA needs exploration [133].

##### Statement #19: Grade of recommendation C-D

In MMA, forced diuresis and alkalinisation of urine with sodium bicarbonate may help to eliminate methylmalonic acid. No data exist in the literature about neuroprotection in acute management in MMA/PA patients.

#### Acute decompensation in known patients with MMA/PA

In MMA and PA, the aim is to stabilize patients on a diet that maintains metabolic homeostasis whilst allowing normal growth and development. Episodes of catabolic stress are associated with rapid production and accumulation of toxic metabolites which can cause decompensation, and lead to life threatening complications. Table 9 shows the triggers, clinical signs & symptoms and common biochemical signs of acute decompensation in MMA/PA. Triggers of acute decompensation include any circumstances inducing catabolism. It is important to recognize the signs and symptoms that indicate the need for intensification of therapy in order to prevent serious complications. The presence of any one or more of such clinical signs and symptoms compared to the individual patient’s base line should trigger further evaluation and potential adjustment of therapy and monitoring in order to prevent complications.

**Table 9:** Triggers, clinical signs & symptoms and biochemical signs of acute decompensation in MMA/PA*

**Triggers**	**Clinical signs and symptoms**	**Biochemical signs**
Infection	Poor feeding	Metabolic acidosis (pH <7.3, anion gap >20 mmol/l, low pCO2 or base excess greater than -5 mmol/l)
Fever	Vomiting	
Prolonged fasting	Lethargy	Elevated blood lactate (>3 mmol/l)
Medication (e.g. chemotherapy, high dose glucocorticoids)	Hypotonia	Hyperammonemia
Prolonged or intense physical exercise, surgery and/or general anesthesia	Irritability	Ketonuria (greater than trace in infants or greater than + in children)
Acute trauma, significant hemorrhage	Respiratory distress	Uric acid and/or elevated urinary urea (urea/creatinine > 20) as signs of catabolism
Psychological stress	Hypothermia	Neutropenia
Excessive protein intake	Dehydration and weight loss	Thrombocytopenia

*Please note that columns are independent from each other. Thus a given line in a column does not refer to the line in the neighboring column.

*Grade of recommendation: D.*

##### Statement #20: Grade of recommendation D

The presence of any one or more of the clinical or biochemical signs listed in Table 9 compared to the individual patient’s baseline should trigger further evaluation and potential adjustment of therapy and monitoring in order to prevent complications.

It must also be noted that there are a few well-documented cases of complications (such as basal ganglia damage) following acute illnesses without biochemical disturbances. Therefore, any intercurrent illness in MMA and PA must be treated as a potential trigger for serious and potentially fatal complications regardless of additional signs and symptoms.

##### Statement #21: Grade of recommendation D

Any acute intercurrent illness must prompt closer monitoring and evaluation.

For emergency management in the hospital see Tables 6, 7 and 8 above.

#### Indications and courses for intravenous fluid therapy

The purpose of IV fluid therapy is to provide sufficient calories to reverse or prevent catabolism as well as fluids and electrolytes when enteral feeding is not possible.

Initial IV therapy with 10% glucose with added electrolytes does not contain protein and the caloric intake provided is insufficient to maintain homeostasis in the long term. Therefore, IV fluids therapy with glucose and electrolytes should not be used for more than 24-48 hours. If enteral protein-containing feeds cannot be reintroduced within this time, parenteral nutrition should be commenced.

Indications for IV fluid therapy in the acute setting may include:

- Any acute presentation with vomiting
- Intolerance of emergency diet given enterally
- Refusal of feeds if nasogastric feeds or gastrostomy are not available/possible
- Suspected pancreatitis or gut pathology
- Prospective management of surgery
- Post operatively during reintroduction of feeds

Some patients require a central line to maintain adequate access during illness; this needs to be carefully weighed against the risk of infection. In the only report available on individuals with central lines, all three required removal for infection [134].

##### Statement #22: Grade of recommendation D

Because of the risk of infection, determination of the need for central lines should be approached with caution and on a patient to patient basis.

#### Nutritional composition of home emergency feeds

During mild illness and without gastrointestinal symptoms, energy intake is often suboptimal and resting energy expenditure may be increased by 30% to 40% during acute decompensation [135]. To prevent acute decompensation home enteral emergency feeding is appropriate. The aim is to provide adequate energy to meet increased metabolic demands and to prevent endogenous protein catabolism. Table 10 gives the nutrient composition of an emergency feed regimen based on a glucose polymer (±long chain fat; note that fat tolerance may be poor during illness) for patients with MMA and PA. It may be necessary to administer suitable enteral feeds continuously via a gastrostomy or nasogastric feeding tube to ensure feed tolerance. Caregivers require appropriate training to conduct this [136]. The minimum amount of energy required to reverse catabolism varies between individual patients and according to the severity of the illness. Pre-measured sachets of emergency glucose polymer have been shown to improve reliability of emergency feed preparation [137]. It is commonly advocated to temporarily stop or reduce natural protein intake but this should be for minimal duration only, and reintroduced within 24(-48) hours to prevent potential catabolism from protein deficiency. The role of MMA/PA precursor-free amino acids during acute management is controversial; it is thought that they may help to minimize catabolism. However, MMA/PA precursor-free amino acids will also increase the feed osmolarity, thereby potentially increasing emergency feed intolerance; moreover, they should be avoided with hyperammonemia (Table 10).

**Table 10:** **Nutritional composition of home emergency feeds in infants and children**

**Age**	**Protein per 100 ml**	**Glucose polymer concentration**	± fat emulsion*	**Energy**	**Energy**	**Suggested daily intake**	**Feeding frequency**
		**% carbohydrate**	**% fat**	**kcal per 100 ml from CHO + fat**	**kJ per 100 ml**	**ml//kg**	
Up to 12 m	Stop or reduce total protein intake by ≥50% depending on illness severity	10	3.5	72	302	120 -150 ml/kg	Continuous tube feds using enteral feeding pump
1-2 y		15	5	105	441	1200 ml	
2-9 y		20	5	125	525	Estimated as indicated	
>10 y		25	5	145	609	Estimated as indicated	

For children >10 kg emergency regimen fluid requirements can be calculated as:

11–20 kg: 100 ml/kg for the first 10 kg, plus 50 ml/kg for the next 10 kg.

>20 kg: 100 ml/kg for the first 10 kg, plus 50 ml/kg for the next 10 kg, plus 25 ml/kg thereafter.

up to a maximum of 2500 ml/day.

*Fat emulsion (50%) is based on a long chain fatty acid source. Fat may not be well tolerated during illness and so may be omitted from emergency feed.

*Grade of recommendation: D.*

Additional water is required particularly during intermittent febrile illnesses and with increased stool losses. In MMA, dehydration with loss of sodium and potassium is a common problem particularly in the presence of renal disease and polyuria. Renal function commonly deteriorates during acute decompensation. Fluid and electrolyte intake require careful management.

Caregivers should not commence an emergency feed without consultation with the inherited metabolic disease (IMD) team. Because the clinical condition may rapidly deteriorate, caregivers require regular contact with the IMD team for assessment of symptoms, energy and fluid intake. For vomiting, diarrhea or any signs of clinical deterioration, patients should be assessed and treated in hospital (see above). Prolonged and frequent use of emergency feeds may lead to protein deficiency.

##### Statement #23: Grade of recommendation D

During mild illness and without gastrointestinal symptoms, home enteral emergency feeding management is appropriate. There should be provision of adequate energy to meet increased metabolic demands and prevent endogenous protein catabolism. Regular review should be conducted by the inherited metabolic disease team.

#### Treatment of fever

Since fever is one of the main factors that can trigger a metabolic decompensation, it is essential to start immediate treatment with antipyretics (e.g. paracetamol, ibuprofen) when body temperature exceeds 38°C.

#### Laboratory investigations to guide acute treatment

Acute treatment is mainly based on commonly available laboratory investigations. Ammonia, acid-base balance and anion-gap are important biochemical parameters that may help to identify an impending metabolic decompensation [138]. Other tests should include urine ketones, glucose, electrolytes (Na, K, Cl, phosphate and HCO_3_), lactate, creatinine, urea, uric acid, albumin, amylase and lipase, and blood cell count. Since these patients are often immunocompromised, blood cultures and CRP should be considered. Specific laboratory investigations include amino acids in plasma/serum to evaluate nutritional supplementation. Additional tests (organic acids, namely methylmalonic acid, propionylcarnitine and free carnitine) are of little benefit in acute management, provided that the diagnosis is already established.

##### Statement #24: Grade of recommendation D

Specific investigations beyond routine tests and amino acids in plasma/serum are of limited help in guiding acute treatment, provided that the diagnosis is already established.

##### Statement #25: Grade of recommendation D

Unless the confirmation of the diagnosis with MMA/PA is still pending, no banking of samples during acute treatment is required except for research purposes.

### Standard long-term management of MMA/PA

The goals of long-term management are to achieve normal development and to prevent episodes of metabolic decompensation, whilst providing good quality of life and avoiding side-effects and complications [139]. Standard therapy includes:

- L-carnitine
- antibiotics to reduce intestinal flora
- vitamin B_12_ in responsive MMA patients
- low-protein diet
- precursor-free amino acid and/or isoleucine/valine supplementation
- vitamin and mineral supplementation
- caring for special situations and provision of emergency regimen in intercurrent illnesses

A detailed, written day to day treatment plan and emergency regimen (see home emergency feeds above), including instructions on when and how to contact the metabolic team or the local hospital should be given to parents/caregivers and to the child’s nursery or school.

#### Statement #26: Grade of Recommendation C-D

The most common medical treatments besides the diet used in long-term treatment of MMA/PA are L-carnitine, antibiotics to reduce intestinal flora and vitamin B_12_.

#### Statement #27: Grade of Recommendation D

Regular follow-up visits to the general pediatrician are recommended. Patients with MMA/PA should receive all regular vaccinations including vaccinations against influenza and rotavirus. Early antipyretic treatment (>38°C) should be given.

#### Pharmacotherapy for long-term treatment

L-carnitine enhances propionyl group elimination [140], regenerates CoA and transforms toxic CoA esters into less toxic carnitine esters [141] that can be eliminated in urine [142-144]. Supplementation restores plasma carnitine levels [42,145]. L-carnitine seems to contribute to the reduction of hyperammonemia in PA patients [118] and demonstrates antioxidant capacity [146]. It is a well-tolerated treatment with few side effects including transient nausea and vomiting, abdominal cramps, diarrhea, fishy body odor. No risks related to high levels of free and total carnitine have been reported. The recommended doses for L-carnitine vary from 100 to 300 mg/kg/d [4,11,121,139]. It is recommended that plasma free and total carnitine levels be regularly monitored to assess compliance and to optimize doses.

##### Statement #28: Grade of recommendation C-D

L-carnitine (100-200 mg/kg/d in 2-4 doses) is useful in the long-term treatment of patients with MMA and PA. Doses should be adapted according to clinical response and carnitine levels.

Metronidazole greatly reduces the production of propionyl-CoA derived from anaerobic bacterial fermentation of carbohydrates in the gut, which may account for a large proportion of total body propionate [147,148]. Intermittent courses of metronidazole may be as effective as continuous treatment [149,150]. Metronidazole, 10–20 mg/kg/day divided in 2-3 times alone or alternating with other antibiotics (e.g. amoxicillin or cotrimoxazole) should be used [4,121,139,151]. In order to avoid the development of drug-resistant colonies, 1-2 weeks of therapy alternating with 2-3 weeks off or alternating every month is advisable. It may be useful to supplement probiotics (avoiding those containing propionic acid producing bacteria) to restore and balance intestinal flora.

##### Statement #29: Grade of recommendation C-D

The use of oral antibiotics continuously or intermittently to control intestinal propionic acid producing bacteria is useful in patients with MMA/PA. The most frequent dosing schemes use metronidazole (10–20 mg/kg/d in 2-3 doses) for 1-2 weeks alternating with 2-3 weeks off or alternating every month with other antibiotics.

In MMA, long-term prognosis correlates with vitamin B_12_ (cobalamin) responsiveness [25,26,92], which has always been found in cblA patients, less commonly in cblB and mut^−^ patients and almost never in mut° patients. Every patient should be tested carefully using a standardized protocol [1] to avoid misclassification of patients with a mild response such as some cblB patients [25]. Hydroxocobalamin is preferred over cyanocobalamin [1,11]. Doses range from 1 to 14 mg/week (IM or IV) and 5 to 21 mg/wk (oral). Parenteral treatment should be tried first followed by combined parenteral/enteral treatment or oral-only treatment depending upon the biochemical response.

##### Statement #30: Grade of recommendation C-D

Response to vitamin B_12_ should be assessed in every MMA patient. For responders hydroxocobalamin should be used as long-term treatment. Doses of hydroxocobalamin have to be tailored individually depending on the clinical and biochemical results.

Growth Hormone (GH) therapy has been used in patients with MMA/PA showing clinical and biochemical improvement, probably due to an anabolic effect [47,74,152]. However the effect appears to be lost at higher dosage. GH may also be of benefit in the treatment of chronic kidney disease in MMA, although no specific studies are available. A lipolytic effect is also seen but seems not to be detrimental on metabolite excretion [153].

##### Statement #31: Grade of recommendation C-D

Growth hormone (GH) has been used in patients with MMA/PA displaying severe growth retardation associated with an abnormal response to GH stimulation tests. Careful monitoring of metabolic parameters under GH therapy is required due to its potential lipolytic effects.

Among the ammonia scavengers, sodium benzoate (150-250 mg/kg per day) has been used for the long-term treatment of chronic hyperammonemia in MMA/PA patients [139,154]. There is no clear evidence of the benefits of this therapy in the chronic setting. Sodium-phenylbutyrate is not advisable because it lowers the glutamine/glutamate ratio. N-Carbamylglutamate was shown to enhance urea genesis in patients with PA suggesting a potential role in treating hyperammonemia in PA patients [155]. However there is no clinical evidence for long-term use of this therapy in MMA/PA.

##### Statement #32: Grade of recommendation C-D

Chronic hyperammonemia indicates metabolic imbalance and requires investigation and treatment of the underlying cause. Sodium benzoate has been used to treat long-term hyperammonemia in MMA/PA patients.

#### Drugs to be avoided

Steroids administered by a systemic route should only be used in emergency situations in patients with MMA/PA due to their catabolic effects on the muscle. However, in exceptional cases such as West syndrome, the management of MMA with concurrent steroid therapy is possible and beneficial [156]. Inhaled steroids are devoid of catabolic effects and seem safe for MMA/PA patients.

##### Statement #33: Grade of recommendation D

Steroids administered by a systemic route should be avoided if possible, or if unavoidable, should be used with caution. Inhaled steroids seem safe.

#### Contraindicated drugs

Drugs containing pivalic acid (antibiotic) and valproate decrease L-carnitine concentration in plasma and tissues by urinary excretion of acylcarnitine as pivaloylcarnitine and valproylcarnitine respectively [157]. Sodium valproate should be used with great caution due to its interference with intermediary metabolism unless there are no other antiepileptic drug alternatives. Nephrotoxic drugs should be avoided in patients with MMA due to their potential to precipitate or aggravate renal disease. Immunosuppressive drugs (e.g. cyclophosphamide) should be used with caution. Medications known to prolong the QTc-interval (such as prokinetic drugs) should be avoided if possible.

##### Statement #34: Grade of recommendation D

Drugs containing propionate, valproate, pivalic acid, nephrotoxic drugs and chemotherapy agents should be avoided or used with great caution in patients with MMA/PA. Medications known to prolong the QTc-interval (such as prokinetic drugs) should be avoided if possible.

### Dietary management of MMA/PA

#### Low protein diet

The basic principles of dietary management are similar for MMA and PA patients. The mainstay of nutrition therapy is a low protein intake, limiting but ensuring essential requirements of the propionic acid precursor amino acids, isoleucine, valine, methionine, and threonine to reduce elevated concentrations of metabolites [151]. The amount of natural protein prescribed is determined by age, growth, metabolic stability and severity of condition. Ideally, when using exclusively natural protein, the FAO/WHO/UNU (2007) safe levels of protein intake should be the ultimate aim (Table 11). Many but not all centers provide additional precursor-free amino acids that supplement natural protein intake in order to achieve protein requirements [11,121]. The source of natural protein is important. If only cereal and vegetable protein sources (low biological value) are consumed, additional protein may be required to compensate [158]. Protein intake should be evenly distributed throughout the day. Patients with mild forms of MMA/PA may tolerate a natural protein intake that is equal to or exceeds the FAO/WHO/UNU (2007) safe levels of protein intake. Occasionally it has been reported that natural protein is enhanced with both precursor-free amino acids and additional single isoleucine and valine [30] supplements but no studies have reported the safety and efficacy of such an approach. Careful monitoring of plasma amino acids, in particular branched chain, are thus required.

**Table 11:** **FAO/WHO/UNU 2007 safe levels of protein and energy intake for different age groups**

**Energy requirements**					Protein requirements*	
**Age**	**kJ/kg/day**		**kcal/kg/day**		**Age**	**g/kg/day**
	**FAO/WHO/UNU 2007**		**Converted from FAO/WHO/UNU 2007**			
**Infants (y)**	Males	Females	Males	Females	Infants (y)	
0.5	335	340	80.0	81.2	0.1	1.77
					0.2	1.5
					0.25	1.36
					0.5-1	1.31
**Children (y)**					Children (y)	
2.5	348	334	83.1	79.8	1-10	0.84-0.90
5.0	315	305	75.2	72.8		
10	275	248	65.7	59.2		
15	230	193	54.9	46.1	11-16	0.92-1.14
**Adults (y)**					Adults (y)	
(Moderate activity, 70 kg)						
18-29	183	159	43.7	38.0	>16	0.84-0.87
30-59	175	148	41.8	35.3		
**Adults (y)**						
(Moderate activity, 50 kg)						
18-29	212	180	50.6	43.0		
30-59	212	183	50.6	43.7		

*The FAO/WHO/UNU (2007) have set safe levels of protein intake titrated as an age adjusted mean + 2 SD. Values for safe levels of protein intake apply to males and females.

##### Statement #35: Grade of recommendation C-D

Dietary management of MMA/PA aims at metabolic stability and normal growth. Protein tolerance should be titrated individually. It is based on adequate energy supply combined with avoidance of prolonged fasting and reduced intake of precursor amino acids through a restricted natural protein diet, commonly supplemented with precursor-free synthetic amino acids. The FAO/WHO/UNU (2007) safe levels of protein intake provide a useful guide for protein prescription.

#### Amino acid supplements

Although supplementary, precursor free amino acids are commonly used to contribute to the total protein intake; their efficacy has not been fully assessed [151] and the amount prescribed in cross sectional and cohort studies varies between 15-50% of total protein intake [11,23,25,151,159,160], partly influenced by metabolic stability, natural protein intake tolerated, patient age, disorder severity and local practice. There is debate about the amount of any extra precursor free amino acid that should be prescribed to account for any inefficiency in its absorption and catabolic rates. In other amino acid disorders, allocation of an extra factor of 20% is given to compensate for ineffective amino acid utilization [161].

##### Statement #36: Grade of recommendation C-D

MMA/PA precursor free amino acid supplements should form part of the total protein intake if natural protein tolerance is below FAO/WHO/UNU (2007) safe levels of protein intake and thereby make up any protein deficit to meet requirements.

#### Energy requirements

Little is known about energy requirements in MMA/PA. Whilst this should be individually determined, there should be a balance between preventing catabolism and overfeeding, particularly if there is decreased physical activity. The FAO/WHO/UNU (2007) recommendations can be used to guide energy requirements (Table 11). Overweight has been reported in MMA and PA children despite energy intakes lower than recommended for age [162]. There have been conflicting reports on resting energy expenditure (REE) in MMA [163-165]. In contrast, energy requirements during illness are increased coupled with an increase in resting energy expenditure [135].

##### Statement #37: Grade of recommendation C-D

Intake of energy is initially guided by energy requirements for normal healthy children but it should be adjusted for age, gender, mobility, physical activity and clinical condition of the child. During metabolic decompensation or intercurrent illness, especially with fever, energy requirements are increased and additional energy supply should be provided.

#### Practical aspects of dietary management

There are few published reports of successful demand breast feeding in MMA/PA [166-168] and some do not advocate this in MMA/PA [169]. Expressed breast milk should be encouraged if demand breast feeding is impracticable. For MMA/PA particular breast milk advantages include its low protein and amino acid content, protection against infection, and reduction in gut propionate.

##### Statement #38: Grade of recommendation C/D

Breast feeding or breast milk with or without MMA/PA precursor-free amino acids may be considered in the dietary treatment of newly diagnosed neonates/infants.

In bottle fed infants with MMA/PA, natural protein requirements are met from measured amounts of standard whey dominant infant formulas. To ensure nutritional requirements are fully met, supplementary precursor-free infant amino formula or protein-free infant formula is used to provide precursor-free amino acids and/or energy and micronutrients.

Weaning, usually with fruits and vegetables, is commenced at the usual age. Due to the development of potential feeding problems, it is important that early feeding is supported by speech and language therapy in addition to dietary care.

In infants with severe phenotypes, tube feeding is commonly used early on to deliver the majority of nutritional requirements [159,160] and prevent nighttime fasting catabolism. Although there are no controlled studies to support this, it has been shown to have a positive effect on morbidity [30,159]. Tube feeding has several advantages [30] as it

- Helps ensure optimal nutritional intake.
- Overcomes severe anorexia and feeding difficulties, which commonly occur within the first year of life.
- Prevents prolonged fasting with release of propiogenic odd chain fatty acids derived from lipolysis [170,171].
- Ensures even distribution of natural protein and energy intake over 24 h.
- Helps to administer medications.
- Permits delivery of prescribed emergency feeds.
- Allows home management of minor illnesses and decreases hospital admissions.

If tube feeding is needed in the long-term, gastrostomy is recommended [172]. Enteral feed composition is usually complex, requiring an individualized protein and energy profile commonly consisting of separate (or combined) modules of sources of protein, fat, carbohydrate, vitamins and minerals. It is essential that all caregivers administering enteral feeding receive professional instruction in safe feed production and that their feed preparation technique is reviewed on an annual basis [136,172-174].

##### Statement #39: Grade of recommendation C-D

Tube feeding may be necessary to avoid catabolism/prolonged fasting, achieve nutritional adequacy, administer medications and supplements and maintain metabolic stability. Continuous training of parents and health care professionals to prepare and administer tube feeding is necessary to minimize safety risks such as incorrect tube position, dislodgement or wrong feed preparation.

Feeding difficulties are commonly reported in MMA and PA [173]. It is estimated that about 55% of MMA children had intermediate to major feeding problems [151] at three years of age. Chronic anorexia, oral hypersensitivity, dysphagia and hyperactive gag reflex are commonly seen. As there are commonly feeding safety issues and delays in feeding development, early assessment, advice and support from a speech and language therapist is important.

##### Statement #40: Grade of recommendation C-D

Feeding problems are common in children affected by MMA and PA. Therefore referral to a special feeding clinic with access to a speech and language therapist may be beneficial.

Over restriction of natural protein can result in poor weight gain, poor growth and poor wound healing. Eight individuals with MMA or PA have been reported with acrodermatititis enteropathica-like skin complications, which were associated with low levels of isoleucine. Consequently it is necessary to ensure adequate levels of isoleucine in feeds [152,175-177].

##### Statement #41: Grade of recommendation B-D

Dietary treatment requires careful nutritional supplementation with clinical, biochemical and dietary monitoring to prevent nutritional imbalance. Provision of adequate isoleucine/branched chain/essential amino acids is needed with the aim of maintaining essential amino acid levels within in the normal range for the local laboratory.

Patients with organic acidemias are at risk of osteopenia and osteoporosis [30,31] which is aggravated as a result of renal dysfunction in MMA. The metabolic diet used in MMA and PA may be low in calcium and vitamin D levels both of which are essential for good bone health. Therefore, it is important that a complete vitamin and mineral supplement is given if dietary requirements are not achieved from natural protein sources and precursor-free amino acid supplements. Single case reports of other nutritional deficiencies have been described [178-181].

##### Statement #42: Grade of recommendation C

MMA/PA patients are at increased risk for osteoporosis. Recommendations for bone health include optimizing nutrition, ensuring adequate calcium and vitamin D. Baseline DEXA is recommended at 10 years and follow-up according to bone-health status. Extra attention should be paid to MMA patients with chronic kidney disease.

Vomiting is a frequent symptom of MMA/PA and may contribute to metabolic instability or aggravate anorexia. Central antiemetic agents such as ondansetron or chlorpromazine may be used with caution. Prokinetic drugs may contribute to prolonged QTc interval. Constipation is another chronic manifestation in MMA/PA patients that should be treated promptly as it has been shown that enhancement of gut motility can improve metabolic stability in patients with PA [182].

##### Statement #43: Grade of recommendation D

Vomiting and constipation are common problems in MMA/PA. They may contribute to metabolic instability and should be anticipated and treated.

#### Metabolic follow-up, monitoring of diet and nutritional status and monitoring of long term complications

MMA/PA patients require lifelong monitoring by the entire metabolic team. Clinical, nutritional, biochemical, neurodevelopmental and psychological assessment should aim at optimizing patient development and performance with age-adapted dietary and drug treatment. Regular monitoring of metabolic parameters, growth, along with measures of protein nutrition and overall nutritional status, as well as regular monitoring for long term complications are indicated (Table 12). Intervals between visits should be decided individually on the basis of age, growth, severity, metabolic stability and compliance with diet and therapy.

**Table 12:** **Metabolic follow-up, monitoring of diet and nutritional status, and long term complications**

**Assessment**	**Frequency**
**1. Metabolic follow-up**	
NH_3_, blood gases, lactate	Each clinic visit
Quantitative plasma amino acids (3-4 h fasting)	Every 3-6 months
MMA plasma and urine	Every 3-6 months
Free carnitine plasma (or dried blood spots)	Every 6-12 months
**2. Monitoring of diet and nutritional status**	
Diet history	Each clinic visit
Growth (weight, length/height, BMI)	Each clinic visit
Clinical examination e.g. skin, hair	Each clinic visit
Albumin, pre-albumin	Every 6 months
Bone health (calcium, phosphorus, alkaline phosphatase, magnesium, parathyroid hormone, 25-OH vitamin D)	Annually, more frequently in case of chronic kidney disease
FBC, zinc, selenium, ferritin, folic acid, vitamin B_12_	Annually
**3. Monitoring of long term complications**	
Neurological examination with detailed history of developmental milestones	Each clinic visit
Kidney function (glomerular and tubular function)* (serum creatinine, urea, electrolytes, cystatin C, uric acid, urinary electrolytes and protein loss, GFR)	Biochemistry, urine: every 6 mo* GFR*: annually, beginning at 6 y or earlier, if other renal function markers are abnormal
Pancreas (amylase &lipase)	Every 6 months
Heart (ECG, echocardiography)	Baseline ➔ annually, start at 6 y
Formal developmental/IQ assessment	At defined ages
Ophthalmologic assessment	Annually after 6 y
EEG, MRI, formal hearing test	If clinical suspicion/indication
Dentist/oral care	Regularly

*Monitoring of kidney function should be performed every 6 months in MMA; in PA annual monitoring is sufficient and GFR measurement is only indicated if other renal function markers are abnormal.

*Grade of recommendation: D.*

#### Management of MMA/PA patients during surgical procedures or prolonged fasting

During general anesthesia or any prolonged fasting it is important to follow an appropriate protocol, minimizing catabolism by providing adequate amounts of calories (Table 13).

**Table 13:** **Considerations and management of general anesthesia**

***Elective procedures***	***Emergency operations and major procedures (>30 minutes)***
- Check that the child is healthy 48 hours before surgery. If not, postpone the operation.	- Seek specialist advice
- Last metabolic work-up must have taken place less than 3 months	- On admission
	• Check plasma ammonia, pH, blood gases
	• If ammonia >100 μmol/l, pH < 7.30 or base deficit > 10 mmol/l) or the child is unwell, cancel elective procedures and/or seek specialist advice
***Management***	
- Stop feeds according to minimal anesthetic requirements and replace by clear carbohydrate containing fluids or intravenous 10% glucose and appropriate electrolytes at a rate allowing to block lipolysis:	
• 8-10 mg/kg/min for neonates and infants	
• 6-7 mg/kg/min for children	
• 5-6 mg/kg/min for adolescents	
• 4-5 mg/kg/min for adults	
- For B_12_ responsive patients administer hydroxocobalamin 1mg parenterally 24 h before and on the day of the procedure	
- Add intravenous L-carnitine: 100 mg/kg/ day (max. 12 g for adults)	
The infusion and treatment should be maintained during the whole surgical procedure. The use of intravenous lipid solution may be considered for longer procedures (1 to 2 g/kg/day IV).	
***Post-operative management***	
- Following the operation, feed depending on metabolic stability at the time you would feed any other patient following an equivalent procedure.	
- Give whatever oral medicines the patient may be due at the same time	
- Discontinue the intravenous infusion ONLY after the child has been seen to tolerate food.	
- Remove the cannula ONLY when normal feeding has been achieved.	
- If recovery is delayed or complicated by vomiting:	
• check blood gases, electrolytes and ammonia	
• consider using antiemetic drugs (ondansetron, avoid metoclopramide)	
• continue glucose and L-carnitine infusion and add IV lipids (1-2 g/kg/d)	
• IV amino acids should be added if the patient cannot be fed (0.5-0.8 g/kg/d)	
- Discharge ONLY after full recovery and normal metabolic results. This will often be the following day.	

##### Statement #44: Grade of recommendation D

Patients with MMA/PA, who are usually well controlled, can easily decompensate during surgery, precipitated by a combination of stress and fasting. Elective surgery in these patients is usually best done at a hospital with an on site metabolic unit.

### Organ transplantation in MMA/PA patients

The role of organ transplantation in the treatment of MMA and PA is currently evolving. Transplantation in MMA and PA should be considered in patients with frequent metabolic decompensations where the clinical condition is difficult to stabilize with dietary/pharmacological treatment [183,184]. Earlier, liver transplantation was associated with high mortality rate [185]. This problem has diminished in more recent years and there have been several reports on successful liver transplantations with a drastic decrease of hospital admissions and improvement in quality of life [186-189]. The most important concerns, particularly in MMA are neurologic complications, such as basal ganglia and cerebellar stroke, movement disorders, tremor and sensorineural hearing loss, occurring even after liver or combined liver and kidney transplantation [190-195]. Thus transplantation can only be considered as a symptomatic treatment aiming at improvement of quality of life, but not as a definitive cure of the disease.

#### Liver/combined liver-kidney transplantation in MMA

Solid-organ transplantation, such as single liver (LT) or kidney transplantation (KT), or combined liver/kidney transplantation, has become an effective alternative treatment option in recent decades [192,195,196]. Isolated liver transplantation should be performed early in life to maintain normal renal function [186]. In liver transplanted MMA patients, decreased but not completely corrected plasma and urine levels of methylmalonate have been observed [191,197]. At more advanced age kidney function will likely continue to decline after LT thus necessitating secondary KT [70,192,193,198]. Renal failure is sometimes even accelerated by the use of immunosuppressive drugs after LT (unpublished but shared expert experience). The perioperative treatment needs to prevent catabolism to avoid metabolic decompensation. Lactate seems to be the most reliable parameter for control [183]. MMA and methylcitrate levels in CSF samples after transplantation remained high [191,193], most probably explained by the presence of the metabolic defect also in the central nervous system (CNS) [199]. Patients thus remain at risk of developing acute or chronic neurological complications after transplantation. It is yet not clear if the continuation of restricted protein intake and medication can prevent these complications.

#### Liver transplantation in PA

LT in PA has been proposed to minimize the risk of further decompensations and to improve the quality of life [12]. However, experience with LT in PA is still limited. Recent case studies have reported clinical improvement, including significant decrease of episodes of metabolic decompensation, better feeding, and improved neurological development after LT [187,200]. Moreover, LT has been shown to effectively reverse cardiomyopathy [44,201]. The effect on the central nervous system remains unclear. There is one report on improved EEG after LT [202], but basal ganglia strokes have also occurred after LT [184,203]. The observation of severe metabolic acidosis 3 years after successful LT without protein restriction [204] and a less significant reduction of circulating metabolites after LT and increased protein intake [205] suggest that a certain level of protein restriction and carnitine supplementation may be indicated even after LT.

##### Statement #45: Grade of recommendation C-D

Liver and/or kidney transplantation should be considered as an alternative therapy to conventional medical treatment in MMA and PA patients with frequent metabolic decompensations where the clinical condition is difficult to stabilize. Ideally, it should be performed before appearance of severe neurologic damage and under stable metabolic conditions. However, transplantation only partially corrects the enzymatic defect; renal and neurological complications may still occur afterwards. Due to the persistent risk of neurological degeneration and/or metabolic decompensation and in the absence of scientific data, maintenance of protein restriction and L-carnitine supplementation after transplantation seems to be warranted. Any indication for transplantation must be decided on an individual patient basis taking into account the balance between expected improvement of life quality and the morbidity/mortality risk related to the procedure.

#### Kidney transplantation in MMA

Single (cadaveric or living donor) KT with a good graft function and metabolic control has also been described for patients with mut^−^, cblA and cblB defects [206,207]. Surprisingly, these patients showed a significant improvement in quality of life with less metabolic decompensations and reduced plasma levels of pathological metabolites after KT [206,208-212]. Since it has been observed that urinary MMA excretion decreased even more significantly after KT or combined liver/kidney transplantation than after single LT, it has been suggested that KT not only corrects renal dysfunction but may also be sufficient to ensure partial correction of methylmalonyl-CoA mutase activity to prevent metabolic decompensation. Thus, it seems that the small amount of enzyme activity gained by KT is sufficient to improve the metabolic balance of MMA patients. Despite some postoperative complications after transplantation [213], the outcome of single LT has been associated with a higher mortality and its effectiveness was hampered by severe post-transplant complications [190]. KT might thus be a safer and more satisfactory treatment option for MMA patients. As to the time point when to perform kidney transplantation, it should be noted that a decrease of urinary excretion of MMA (even erroneously interpreted as a stabilization of metabolic control) and an increase of plasma concentrations is often seen before end stage renal disease is reached. This results in an increased risk of neurologic damage and further impaired kidney function. The decision for KT should be individualized and made jointly between the metabolic and nephrology teams.

##### Statement #46: Grade of recommendation D

For MMA patients in end stage renal failure combined liver/kidney transplantation has been mostly used so far. However, several reports on isolated kidney transplantation evoke this procedure as an alternative and safer strategy as it seems to restore sufficient enzyme activity and also improves the quality of life of patients.

#### Type of liver transplant, donor and ethical issues

Standard orthotopic liver transplantation (OLT) is preferred to auxiliary LT because it has been associated with fewer complications [197]. Transplantation of liver lobes from living relatives can reduce waiting times and gives results comparable to those obtained with cadaveric organs, albeit with a small risk for donors [197]. Heterozygosity for the disease in the living related donor is not a contraindication. The benefits of organ transplantation must be individually and meticulously weighed against the risk of perioperative complications, including renal and neurological progressive impairment in the post-transplant period. Decisions on whether or not to perform LT entail ethical considerations requiring individualized decision, in particular when the child is already neurologically impaired or when living donor LT is considered [214].

##### Statement #47: Grade of recommendation D

If liver transplantation is considered, the recommended procedure is orthotopic liver transplantation. Ethical issues concerning the risk for the recipient and for living donors make careful pre- and post-transplantation counseling obligatory.

### Experimental therapies

Liver cell transplantation and liver progenitor cell transplantation have been proposed to restore some of the lacking enzyme activity in the liver by infusion of either liver cells or liver progenitor cells [215-218]. Clinical trials testing the safety and efficacy of the procedures in urea cycle disorders are ongoing. Application in patients with other inborn errors of metabolism (including MMA/PA) is planned for a second phase.

Proof of principle for a nonsense read-through therapy in PA [219] and for chaperone therapy in MMA [220] has been achieved in cellular models.

Successful gene therapy has been reported for adeno-associated viral gene delivery in the lethal Mut^-/-^ mouse model [221-223]. No clinical trial has been performed in humans so far.

From a pathophysiological point of view the use of antioxidants to reduce oxidative stress may be indicated in MMA and PA [224-231]. So far evidence for clinical efficacy is lacking and no systematic study or treatment trial has been performed. The choice of antioxidant substance(s) and dosage, particularly in the pediatric age group, remains to be determined.

#### Statement #48: Grade of recommendation D

The administration of antioxidants is potentially beneficial for MMA and PA patients, but needs to be confirmed in prospective clinical studies. The use of liver cell transplantation or liver progenitor cell transplantation is not presently a therapeutic option for MMA and PA patients since no clinical experience is available for these disorders. Treatment strategies based on gene therapy and read-through/chaperone therapy show promising results in preclinical studies and might become interesting options in the future.

### Long-term complications and management

Long-term survival in MMA/PA has significantly improved over the last twenty years [21,25,26]. Therefore long-term complications become increasingly apparent and pose new challenges in patients’ care.

For an overview on metabolic follow-up, monitoring of diet and nutritional status as well as monitoring of long term complications please see Table 12.

#### Cognitive development and health related quality of life

Data on the extent and characteristics of mental development and intellectual disability in both diseases are presented non-uniformly in the literature, i.e. a variety of evaluation instruments and test batteries have been used in different studies. Furthermore, patient populations, as well as cut-off values for normal development were specified differently and patients were tested at different points of time.

In MMA, vitamin B_12_ non-responsiveness [232], early onset of disease [121,159], the presence of hyperammonemia at diagnosis or a history of seizures [58], as well as a mut° phenotype [25,48,233] were associated with more severe cognitive impairment. A significant deficit of processing speed seems to be a general feature [58].

##### Statement #49: Grade of recommendation C

Vitamin B_12_ non-responsiveness, early onset of disease, hyperammonemia at onset, a history of seizures, as well as mut° as underlying cause of MMA are risk factors for significant impairment of cognitive development.

PA seems to be associated with cognitive impairment in a significant number of patients which in most studies exceeds 50% of included patients [23,30,33,121,160,232]. At present it remains unclear whether the early onset form results in more severe impairment of cognitive functions than the late onset form.

##### Statement #50: Grade of recommendation C

PA seems to be generally associated with significant cognitive impairment.

Several prospective and retrospective studies on PA [30,234] and MMA [48,235] indicate that developmental delay and intellectual disability may not be prevented by (early) treatment. This observation is supported by a recent study showing that early diagnosis of PA through newborn screening seems to be associated with lower mortality but does not result in improved neurocognitive development [21].

##### Statement #51: Grade of recommendation C-D

Despite intensive medical treatment, MMA and PA are associated with a high frequency of intellectual disability. Cognitive impairment seems to become pronounced from the second year of life.

#### Monitoring of cognitive development and health-related quality of life

Besides clinical assessment, a wide variety of evaluation instruments and test batteries at different measurement points have been reported in the literature. Thus no recommendation of specific tests or measurement intervals can be deduced.

##### Statement #52: Grade of recommendation D

Intellectual abilities and cognitive development should be assessed early and reliably to allow timely referral for therapeutic and rehabilitative intervention. Testing should be age-appropriate using standardized instruments. Culturally appropriate and language-free methods are recommended for patients with impaired speech or command of the language.

##### Statement #53: Grade of recommendation D

Health related quality of life, stress and psychosocial factors are meaningful outcome parameters which have not been investigated systematically in MMA/ PA patients and their caregivers so far, but should be considered and addressed in the future. Standard settings of care in terms of a multidisciplinary approach including social workers, psychologists, dieticians, rehabilitation services and specialized physicians should be defined.

#### Neurological complications

Neurological symptoms are frequent in MMA/PA at the beginning and during the course of the disease (see also Tables 3 and 5). Regarding diagnosis, management and monitoring of long-term neurological complications see also Table 12.

##### Statement #54: Grade of recommendation D

Neurological examination with detailed history of developmental milestones should be a routine part of evaluation in every visit to the metabolic clinic. Input from a pediatric neurologist should be considered in case of acute neurological presentation (encephalopathy/coma, seizures), any suspicion of developmental delay, spasticity/dystonia and movement disorder, epilepsy, hearing or visual field defects.

Seizures/Epilepsy can be an acute or chronic manifestation of MMA and PA. In early childhood seizures tend to be generalized or myoclonic, and during later childhood generalized or absence in nature [24,28,35,139,236,237] Patients should be referred to a pediatric neurologist at the time of a seizure event. Antiepileptic drugs (AEDs) should be introduced according to seizure semiology and EEG findings [24,28,237]. Corticosteroids have been successfully used in the management of a patient with MMA and infantile spasms with a hypsarrythmia pattern on EEG [156].

##### Statement #55: Grade of recommendation D

Epilepsy is common in MMA and PA patients. In patients with clinical suspicion of seizures an EEG should be performed. Referral to a pediatric neurologist is recommended in the case of a seizure event, and/or if epileptiform activity is detected in the EEG. AEDs for epileptic seizures must be chosen according to seizure types and EEG abnormalities. Valproate is not contraindicated but is associated with a risk of hyperammonemia.

Movement disorders are frequent, being reported in 19-45% of patients with MMA and PA [25,28,33]. L-Dopa/Carbidopa, trihexylphenidyl, clonazepam, baclofen (oral, continuous intrathecal, ventriculoperitoneal shunt) treatments can be effective in patients with acute/chronic dyskinesia and movement disorder [238,239]. For the long term monitoring of patients with extrapyramidal symptoms (dyskinesia, myoclonus, choreoathetosis), and pyramidal tract involvement (spasticity), functional scales such as Barry-Albright scale for dystonia, and Ashworth scale or Gross Motor Function Measure for spasticity may be helpful to objectively monitor patients and effectiveness of treatment protocols [238,240]. A multidisciplinary team involving physical and rehabilitation therapists, and pediatric neurologists should evaluate patients with extrapyramidal symptoms and spasticity [139].

Metabolic stroke-like events involving the basal ganglia have been described in about 10 MMA and PA patients [21,35,55,60-63] and these have usually occurred during or shortly after metabolic decompensation. However, several cases of PA have been published with purely neurologic presentation without the expected heralding ketoacidotic attacks [7,20,35,59,64,65].

Adequate fluid and caloric intake and symptomatic treatment of focal neurological deficits and altered mental status during stroke-like episodes is crucial [139].

##### Statement #56: Grade of recommendation C-D

Movement disorders are frequent in MMA and PA and may be associated with stroke-like events and basal ganglia necrosis. Treatment is symptomatic.

Optic neuropathy has been reported as a late complication in at least fourteen patients with MMA or PA [31,66-69,241]. Acute or chronic visual loss due to optic neuropathy may be a rare complication. Antioxidant drugs have been used to treat optic neuropathy with improved visual acuity reported in one patient treated with high dose vitamin E and Coenzyme Q10 [66]. Consequently, ophthalmologic evaluation by an ophthalmologist including visual acuity as well as visual examination of the anterior chamber, and dilated evaluation of the fundus should be routinely performed [139].

##### Statement #57: Grade of recommendation D

Ophthalmologic assessment (fundoscopy, visual acuity and visual field) and a formal examination by an ophthalmologist to exclude optic neuropathy should be a part of routine evaluation in MMA and PA patients at baseline, at any time of concern and yearly after 6 years of age. Antioxidant drugs have been used experimentally in the treatment of optic atrophy.

Neuroimaging provides information about the timing, extent, reversibility, and possible mechanisms of neural injury, and can be used as a complementary tool to track the course of the disease in MMA and PA patients. Routine neuroimaging in the absence of neurological findings is not necessary [237] and risks of sedation and anesthesia for the imaging procedure should be considered. In a patient with neurological symptoms at presentation and/or any time during the course of the disease, magnetic resonance imaging (MRI) is the preferred imaging technique. MRI sequences should ideally include diffusion weighted imaging (DWI), apparent diffusion coefficient (ADC) maps, axial T2 and FLAIR, sagittal and axial T1.

**Figure 2:**
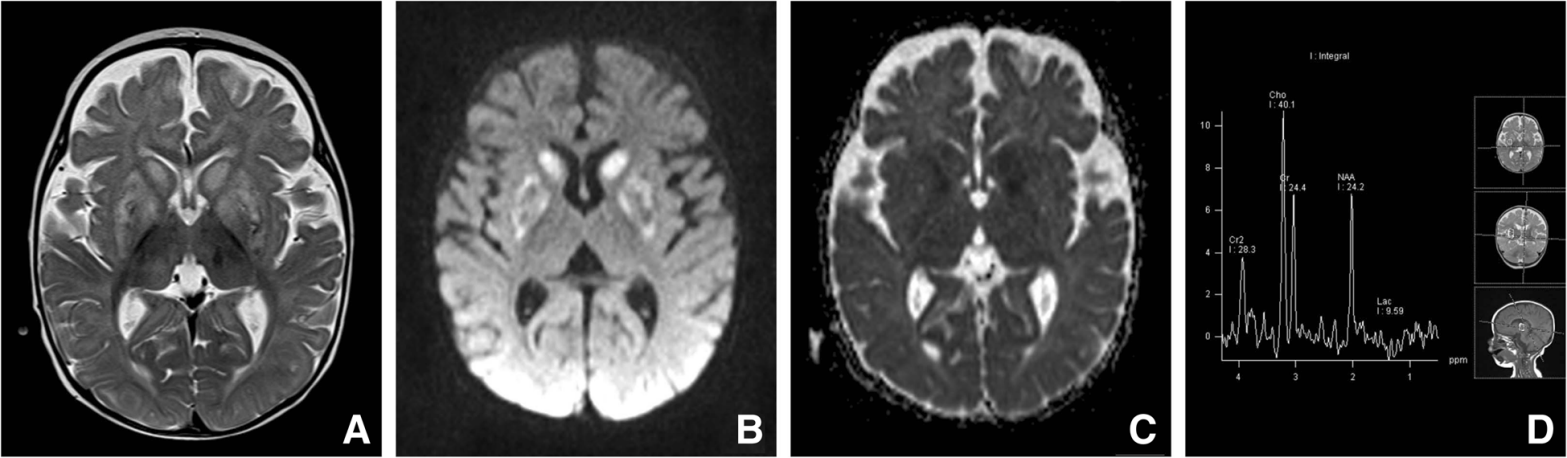
**Neuroimaging finding in MMA: Male child with MMA (cblA defect).** An MRI study was obtained at the age of 6 months because of irritability, feeding difficulties, developmental delay, encephalopathy and metabolic decompensation. The caudate and lentiform nuclei are swollen and hyperintense on axial T2-weighted (W) turbo spin-echo (SE) image **(A)** with restricted diffusion suggested by hyperintensity on trace diffusion-weighted imaging (DWI) **(B)** and low signals on ADC map **(C)**. Proton MR spectroscopy (TE:135ms) performed from the lesions reveals decreased N-acetyl-aspartate (NAA), increased choline (Cho) and presence of lactate **(D)**.

**Figure 3:**
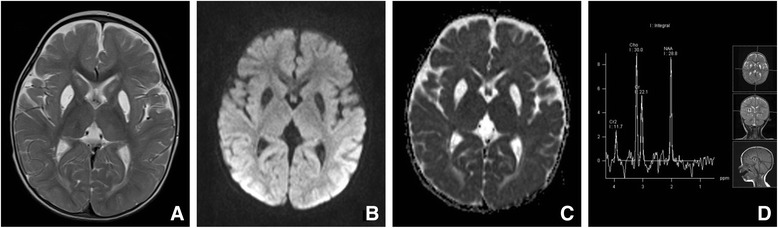
**Neuroimaging finding in MMA: Male child with MMA (cblA defect).** Follow-up MR imaging 6 months after acute deterioration shows residual T2 hyperintensity in the caudate nuclei and necrosis in the lentiform nuclei with reversal of abnormalities in the globus pallidi **(A)**. There is CSF-like unrestricted diffusion in the lentiform nuclei on DWI **(B)** and ADC map **(C)** and elevated Cho on MR spectroscopy **(D)**.

**Figure 4:**
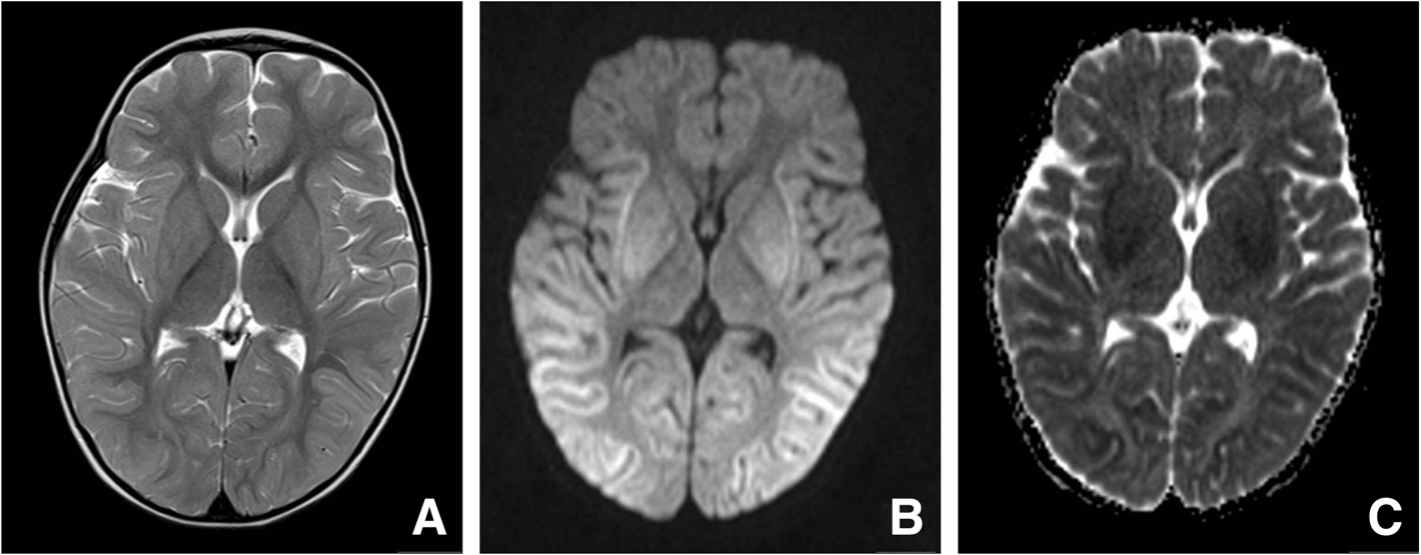
**Neuroimaging finding in PA: Male child with PA, symptomatic from day 4 of life, diagnosed at the age of 4 months.** An MRI was obtained at the age of 5 years when he developed acute encephalopathy. Cerebral cortices especially of the temporal and occipital lobes and the basal ganglia (caudate and lentiform nuclei) are swollen and mildly hyperintense on axial T2W TSE **(A)**. Hyperintensity on trace DWI **(B)** and low intensity on ADC maps **(C)** suggestive of restricted diffusion are seen in those affected regions.

**Figure 5:**
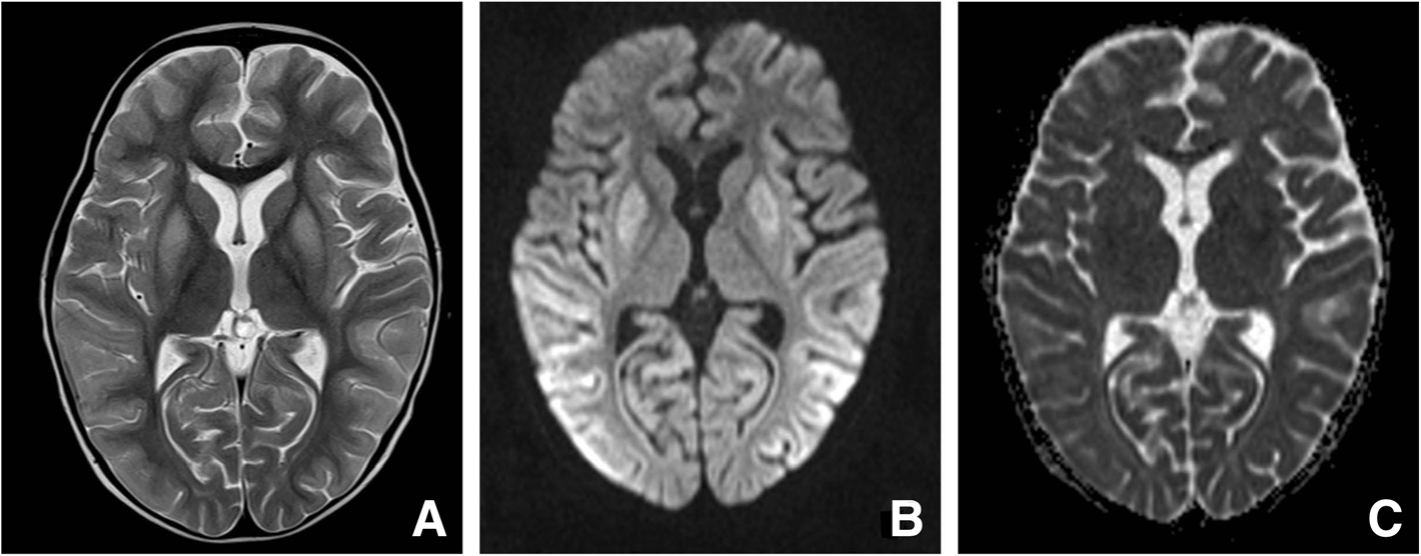
**Neuroimaging finding in PA: Male child with PA, symptomatic from day 4 of life, diagnosed at the age of 4 months.** A week later a repeat MR study showed more intense T2 signal changes in the involved regions, more prominent in the putamina **(A)**. Although a higher signal intensity of the cortices and basal ganglia on trace DWI **(B)**, with disappearance of low signal of the cortices and presence of higher signal intensity on ADC maps **(C)** there’s pseudonormalization of diffusion restriction.

Neuroimaging findings are similar in MMA and PA. Typically, there is symmetric involvement of basal ganglia, with selective pallidal involvement reported in MMA [242]. Edema, sometimes with restricted diffusion suggests a cytotoxic insult and is a well-documented phenomenon in PA in the acute phase, most frequently found in basal ganglia, but also in the cerebral cortex [242,243]. Resolution of findings on routine T2W images, DWI and ADC [60,243,244] usually corresponds to improvement of clinical symptoms and biochemical parameters. Figures 2, 3, 4 and 5 show typical (as judged by the guideline group) neuroimaging findings at the time of acute clinical and/or metabolic decompensation in MMA (Figures 2 and 3) and PA (Figures 4 and 5).

During the first year of life there is moderate to severe widening of sulci and fissures more commonly reported in PA patients. Resolution with treatment is reported in MMA patients, and there is mild to moderate delay in myelination [242,245]. Thin corpus callosum and brainstem atrophy have also been described [246]. It should also be recognized that, the brain parenchymal metabolite profile may be affected without significant metabolic decompensation, and even without structural changes on neuroimaging patients may develop movement disorders [35].

^1H^Magnetic Resonance Spectroscopy (MRS) is a powerful clinical tool as a complementary method to MR imaging to understand disease process in the brain, particularly by *in vivo* demonstration of brain metabolites [245,247]. MRS in acute phases demonstrated decreased NAA and increased lactate peaks, and normalization of values with treatment, in parallel with improvement of clinical symptoms and biochemical parameters [234,247,248]. Abnormal MRS in the presence of negative MRI findings may help to define the region vulnerable and at risk for damage in the CNS [234,245,249].

In the research setting, advanced imaging such as magnetic resonance spectroscopy (MRS), diffusion-tensor imaging (DTI), and functional imaging studies (PET) may provide useful and sensitive complementary information for the assessment of brain damage and follow-up [244,248,250,251].

##### Statement #58: Grade of recommendation D

Routine screening MRI in the absence of changes in neurological symptoms is not necessary and may expose the patient to additional risks related to anesthesia. Conversely, at the time of newly appearing neurological complications MRI may be indicated.

Visual evoked potentials (VEP) should be included in ophthalmological evaluation as a complementary tool since acute or chronic visual loss due to optic neuropathy is possible during the course of the disease (see above). Hearing loss can be a rare complication of MMA/PA [236]. Evaluation of hearing (brainstem auditory evoked response (BAER) and/or audiometric tests) should therefore be performed as indicated.

##### Statement #59: Grade of recommendation D

Evoked potentials (visual evoked potentials: VEP; brainstem auditory evoked responses: BAER or audiometry) should be considered if clinically indicated.

#### Renal complications

All individuals with isolated MMA, even those who are mildly affected, are thought to be at risk of developing renal insufficiency [252]. Chronic kidney disease (CKD) occurred in 47% of patients with a median age of onset at 6.5y [48] and was found most frequently in mut° (61%) and cblB (66%), less frequently in cblA (21%) and mut^−^ (0%) patients [25]. CKD was predicted by the urinary excretion of methylmalonic acid and the disease course was further aggravated by secondary complications of CKD, such as anemia, arterial hypertension, renal osteodystrophy, and hyperparathyroidism [25]. CKD often remained stable for several years, sometimes even until adulthood; progression to end-stage renal disease occurred in 12-14% of patients [25,48]. A recent study reported that the rate of increase of kidney growth significantly decreased in patients with MMA over time and was predicted by height, serum cystatin C, and serum methylmalonic acid concentration [253].

In patients with MMA two main pathologies have been reported, chronic tubulo-interstitial nephritis [49], and renal tubular acidosis [254,255].

On renal biopsy chronic tubulo-interstitial nephritis with mononuclear cell infiltration, interstitial fibrosis and tubular atrophy has been demonstrated [53]. The precise pathogenesis remains obscure, but recent studies suggest that proximal tubular mitochondrial dysfunction and increased serum methylmalonic acid levels are key pathogenic mechanisms in MMA-associated kidney disease [229,230,256].

In PA, renal insufficiency has been reported in 3 patients [27,70,257].

##### Statement #60: Grade of recommendation C

One of the most severe long-term complications in MMA is chronic kidney disease (CKD). The two most frequent complications reported are chronic tubulo-interstitial nephritis and renal tubular acidosis. CKD is characterized by progressive functional abnormality leading to chronic renal failure. The precise pathogenesis remains obscure but proximal tubular mitochondrial dysfunction and increased serum MMA levels have been proposed as key pathogenic mechanisms in MMA-associated kidney disease. In PA renal failure has been reported anecdotally.

#### Management of long-term renal complications

Standard medical treatment and follow-up of CKD follows the established general therapeutic principles of patients without an inborn error of metabolism [258] including control of blood pressure, electrolytes, secondary hyperparathyroidism, and renal anemia and associated osteodystrophy. Experience with renoprotective measures in the pediatric population has not been thoroughly studied. In case of microalbuminuria or proteinuria in association with hypertension, ACE inhibitors or angiotensin receptor antagonists are recommend with the aim of reaching age adjusted targets of blood pressure control [259].

Treatment with oxygen radical scavengers, such as coenzyme Q10, glutathione, MitoQ might be beneficial [230]. Further therapeutic strategies that aim at decreasing toxic metabolites, including dietary restriction of precursor amino acids or an enhanced tubular excretion of toxic compounds, may also protect the kidney. Like for other CKD, growth hormone may also be of benefit, although no specific studies are available.

Renal replacement therapy, including hemodialysis/hemodiafiltration or peritoneal dialysis, is not only important for long-term treatment of end-stage renal disease but also as a preoperative measure before organ transplantation or to treat severe hyperammonemia during acute metabolic crisis, especially in neonates.

Finally, it has been suggested that kidney transplantation not only corrects renal dysfunction but may also provide partial correction of the enzymatic defect and improve metabolic control (see above).

##### Statement #61: Grade of recommendation C-D

The management of the long-term renal complications is based on adequate hydration, drug therapy, hemo- or peritoneal dialysis and ultimately kidney transplantation.

#### Diagnostic tests to monitor renal long-term complications

Renal function should be carefully monitored (see also Table 12) because of the risk of developing end-stage CKD requiring hemodialysis and kidney transplantation [26]. Serum creatinine-based glomerular filtration rate (GFR) estimates may overestimate true GFR due to low muscle mass [46] and low protein intake. Cumulative urinary MMA excretion over time (measured in repetitive spot urines) correlates with the risk of CKD and has been proposed as a follow-up parameter for renal function [25]. However, with declining kidney function urinary MMA ceases to be a reliable marker and should be replaced by plasma MMA. Thus GFR estimates that include serum cystatin C (and creatinine) as well as plasma MMA appear to be the most accurate [253]. Ideally inulin or iohexol clearance should be measured.

##### Statement #62: Grade of recommendation C-D

Regular measurements of glomerular filtration rate and plasma and urinary MMA are recommended as parameters in the follow-up of patients with MMA. With declining kidney function urinary MMA ceases to be a reliable marker and should be replaced by plasma MMA. Plasma creatinine levels may be less reliable in these patients due to the poor muscle mass and the low protein intake and may be complemented with cystatin C, however trends can still be informative.

#### Cardiac complications

Cardiomyopathy [21,23,26,31,39,43,44,71,148,260] and prolonged QTc interval [21,31,38,40,41,56] are emerging as potentially lethal complications in MMA and, more prominently, PA. Additionally there are several reports of heart failure in patients with MMA and PA who did not have cardiomyopathy [187,261,262]. Median age of presentation of cardiomyopathy was 7 years [44]. The etiology of prolonged QTc interval is not well understood but sudden death has been described in this patient population [38,40]. Liver transplantation has been shown to improve cardiomyopathy in 3 PA patients, one of which had severe heart failure [44,261].

##### Statement #63: Grade of recommendation C-D

Cardiac complications include cardiomyopathy and prolonged QTc interval which may be life-threatening and may occur in MMA and PA patients with increasing frequency with age. Therefore ECG and heart ultrasound (echocardiography) are recommended every year. If cardiomyopathy or long QTc is present standard cardiac therapy should be undertaken and metabolic treatment and monitoring should be optimized. Liver transplantation has been reported to reverse cardiomyopathy in PA.

#### Pancreatitis

Acute, recurrent acute and chronic pancreatitis are possible long-term complications of MMA [72,73] and PA [75,76,263]. The pathogenesis is unclear.

##### Statement #64: Grade of recommendation C-D

Acute, recurrent acute and chronic pancreatitis may develop in MMA and PA, independently from metabolic decompensations and metabolic control. Clinical presentation of pancreatitis is similar to individuals without MMA/PA, but can mimic features of metabolic decompensation.

#### Diagnostic tests and investigations to monitor pancreatic long-term complications

(see also Table 12) Similar laboratory changes [72,73,75,76,263] and radiographic signs [72] to those seen in any patient with pancreatitis have been reported including elevated blood glucose, lipase and amylase (may be normal in the beginning) and lowered calcium. Acidosis and ketonuria have also been reported, but not hyperammonemia. Amylase and lipase may be elevated in patients with renal insufficiency and have been reported in a single PA patient during metabolic decompensation but without evidence of pancreatitis on ultrasound [263]. Measurement of lipase and amylase is recommended at annual intervals (especially if chronic renal failure develops) and whenever pancreatitis is suspected. If pancreatitis is suspected MRI/MRCP or CT scan should be performed as recommended in patients without metabolic disease.

##### Statement #65: Grade of recommendation C-D

Diagnosis of acute or chronic pancreatitis follows the same principles as in any other case of pancreatitis.

#### Management of long-term pancreatic complications

Acute pancreatitis should be managed as in any other case of pancreatitis including fluid resuscitation and pain management. Enteral (jejunal) nutrition is preferred [264]. The only specific recommendation for MMA/PA patients has been made for carnitine supplementation in PA patients [139].

##### Statement #66: Grade of recommendation D

Management of acute pancreatitis follows the same principles as in any other case of pancreatitis. Adequate carnitine supplementation is recommended.

*Hematological and immunological complications* are common in MMA and PA. Pancytopenia at initial presentation has been described in a number of patients [21,31,265-270]. Moreover, signs of immunodeficiency, especially neutropenia, are quite common also during the course of disease [7,23,31] and these may resolve with aggressive therapy [31,271]. Isolated thrombocytopenia may occur during any metabolic decompensation [7,21,31,265,272].

There are several reports of infections with numerous types of pathogens and death by sepsis [7,160,266,267,270,273]. Moreover, central line placement has been compromised by infection [134]. There is very little experience with granulocyte colony stimulating factor (G-CSF) and/or intravenous immunoglobulin in this patient population [31]; indication for treatment with G-CSF therefore remains unclear.

Despite a concern about immunological dysfunction, patients with inborn errors of metabolism are not at increased risk of metabolic decompensation following appropriate vaccinations [274] and thus all childhood immunizations should be undertaken. In addition, influenza vaccine should also be given.

##### Statement #67: Grade of recommendation C

Pancytopenia (especially neutropenia) is frequent in MMA and PA and may respond to improved metabolic control. Evaluation with a low threshold to treat infections as well as infection control practices should be performed according to institutional guidelines early in neutropenic patients. Standard immunizations should be given.

#### Risk of cancer and liver cirrhosis

Despite the association of cancers with mutations in genes coding for Krebs cycle enzymes, there is only one case of cancer (hepatoblastoma) in the literature, which occurred in a MMA patient 2 years after a renal transplant and 3 years after growth hormone therapy [213]. To our knowledge, liver cirrhosis has been found in 2 PA patients and in one MMA patient. However these have not yet been reported in the literature.

### Special issues in adult care

#### Women’s health and pregnancy

Pregnancy issues, contraception and sexual health should already be discussed during adolescence [275,276]. No disease-specific contraindications to the use of hormonal contraceptives in MMA and PA patients have been reported. In some cases, hormonal contraceptives may be used to prevent perimenstrual metabolic instability. For patients with functional amenorrhea hormone replacement therapy or hormonal contraceptives should be offered.

Successful pregnancies have been reported in relatively mild cases of MMA (mut^−^ or vitamin B_12_-responsive) [232,275,277-279] and PA [275,280]. These patients remained metabolically stable throughout pregnancy with delivery of healthy children. However, there may be a reporting bias. Based on the few case reports available, methylmalonic acid does not appear to be teratogenic [279], and this presumably holds true for other metabolites accumulating in stably controlled MMA or PA.

Pregnancy should be planned ahead and metabolic control optimized prior to conception.

During pregnancy, the protein demand/tolerance and carnitine requirements may increase and treatment should be adapted accordingly. The carnitine dose should be increased according to measured free carnitine levels. Due to the risk of protein energy malnutrition and micronutrient deficiency, regular nutritional assessment during pregnancy is mandatory. Morning sickness (or hyperemesis) with nausea and vomiting must be taken seriously, as it constitutes a risk of metabolic decompensation and/or protein energy malnutrition, and treatment (e.g. potent antiemetics) should be initiated early.

During labor and delivery there is a higher energy demand and supplemental calories should be supplied to minimize the risk of metabolic decompensation, using intravenous glucose (*e.g.* 10% 2 ml/kg/h = 200 mg/kg/h) [275,276]. Postpartum catabolism constitutes a risk of metabolic decompensation due to a massive internal protein load with the involution of the uterus and thus demands close monitoring and intervention. However, no serious problems have been noted in the reported cases of mild MMA and PA. Breastfeeding also increases energy demands and adequate energy intake should be insured.

##### Statement #68: Grade of recommendation D

Pregnancy should be planned ahead and metabolic control optimized prior to conception. Regular nutritional assessment and metabolic monitoring during pregnancy is mandatory. An increase in protein demand/tolerance and carnitine requirements may occur and treatment should be adapted accordingly.

Supplemental calories, intravenous glucose and carnitine should be supplied peripartum to minimize the risk of metabolic decompensation. Postpartum catabolism constitutes a risk of metabolic decompensation and demands close monitoring.

#### Transition and social issues

Literature specifically addressing social issues and long-term needs of adult patients with MMA or PA is scarce. The majority of MMA and PA patients surviving into adulthood are not able to live independently and need some kind of social support [232]. However, some patients with mild and/or late onset disease and normal psychomotor development may reach independence and higher education. Continuing from childhood to adult life, appropriate services and structures should be provided to support psychosocial development and special educational needs. Transition of a young patient from pediatric to adult services should begin early to prepare a teenager (and their family) for the move to adult services, with encouragement of independence and self-confidence. Adult patients with chronic disease are at risk for developing mood disorders, which may complicate treatment. Depressive disorders should be recognized and appropriate treatment provided.

##### Statement #69: Grade of recommendation D

Transition from pediatric to adult services should begin early and be well coordinated with encouragement of independence and self-confidence. The majority of patients surviving into adulthood need some kind of social support. Services and structures should be provided to support psychosocial development and special educational needs beyond childhood throughout adult life.

#### Recommendations for long-term follow-up (see long term complications above)

Adult patients with MMA or PA have multisystem problems and require coordinated, multidisciplinary care, with emphasis on monitoring and management of long-term complications (*e.g.* renal, bone, cardiac, neurological, ophthalmological, see Table 12). In general, protein tolerance may improve in adulthood and hospitalizations tend to be less frequent [232]. However, prevention of chronic protein-malnutrition and micronutrient deficiencies is essential and regular nutritional assessments are recommended.

Anorexia and feeding problems are frequent in adult patients, with some still requiring nocturnal tube feeding or energy supplements during the day to maintain satisfactory nutritional status. Many have short stature and low bone mineral density. There are no formal recommendations for protein intake in adult patients with organic acidemias. There are reports of protein tolerance increasing and hospitalizations for metabolic decompensation occur less frequently. Many patients may follow a self-restricted protein intake with between 0.5 and 1.1g/kg per day [232].

For osteopenia or osteoporosis, treatment decisions need to be individualized, integrating measured parameters of bone metabolism and including secondary hyperparathyroidism in patients with chronic renal failure. In patients with renal failure, the diagnosis of osteoporosis should be made only in the absence of renal osteodystrophy. The role of antiresorptive drugs (*e.g.* bisphosphonates) for treatment or prevention of osteoporosis has not been studied in MMA/PA; thus, treatment decisions need to be made individually (after excluding renal osteodystrophy). Tools assessing fracture risk (such as FRAX) [281,282] are not designed or validated for this patient group, and therefore are not suitable for guiding treatment decisions. The role of RANKL-Inhibitors (Denosumab) will need to be established in this patient group, but may be an option for patients with osteoporosis and relative contra-indications to bisphosphonates, e.g. renal failure.

Medical treatment of heart failure in patients with cardiomyopathy follows the established therapeutic principles of patients without MMA/PA [283]. However, PA patients should be evaluated for liver transplantation, which has been reported to reverse cardiomyopathy in some PA patients [44].

##### Statement #70: Grade of recommendation D

Adult patients with MMA or PA have multisystem problems and require coordinated multidisciplinary care, with emphasis on monitoring and management of long-term complications (see long-term complications above).

## Closing remarks

These guidelines are the result of a three year process based on the SIGN methodology and are aimed at delivering the best available level of evidence for any given recommendation. The rarity of MMA/PA and the virtual absence of international registries at least until recently, have resulted in mostly C or D levels of evidence for the statements made, which correspond to inferences derived from non-analytical studies such as case reports, case series or expert opinion. Therefore, these recommendations should not be considered infallible or absolute. The working group of this guideline commits itself to revise the work in the future in an effort to preserve the achieved quality and to search for higher evidence levels that might be achieved with time. Indeed, it is hoped that many of the statements will be substituted in forthcoming years by even more precise and effective recommendations to the benefit of the patients.
